# Investigating the ecological fallacy through sampling distributions constructed from finite populations

**DOI:** 10.1515/mcma-2024-2013

**Published:** 2024-08-08

**Authors:** David J. Torres, Damain Rouson

**Affiliations:** Department of Mathematics and Physical Science, Northern New Mexico College, Española, NM 87532, USA; Computer Languages and Systems Software Group, Lawrence Berkeley National Laboratory, Berkeley, California, USA

**Keywords:** Ecological fallacy, sampling distributions, Pearson R, Monte Carlo simulation, linear regression, multiple regression, 62J05, 62H10, 62P25

## Abstract

Correlation coefficients and linear regression values computed from group averages can differ from correlation coefficients and linear regression values computed using individual scores. This observation known as the ecological fallacy often assumes that all the individual scores are available from a population. In many situations, one must use a sample from the larger population. In such cases, the computed correlation coefficient and linear regression values will depend on the sample that is chosen and the underlying sampling distribution. The sampling distribution of correlation coefficients and linear regression values for group averages will be identical to the sampling distribution for individuals for normally distributed variables for random samples drawn from infinitely large continuous distributions. However, data that is acquired in practice is often acquired when sampling without replacement from a finite population. Our objective is to demonstrate through Monte Carlo simulations that the sampling distributions for correlation and linear regression will also be similar for individuals and group averages when sampling without replacement from normally distributed variables. These simulations suggest that when a random sample from a population is selected, the correlation coefficients and linear regression values computed from individual scores will not be more accurate in estimating the entire population values compared to samples when group averages are used as long as the sample size is the same.

## 1 Introduction

Linear regression coefficients, the Pearson *R* correlation, and the coefficient of determination $R^{2}$ have long been used to quantify the relationship between dependent and independent variables. The “ecological fallacy” has shown that linear regression and correlation coefficients based on group averages cannot be used to estimate linear regression and correlation coefficients based on individual scores [14]. Shih, Bradley and Yabroff acknowledge the ecological fallacy in health disparities research [16]. A census-based approach was evaluated by Geronimus and Bound [6]. Many have proposed methods to infer individual-level relationships from aggregate data [7, 1, 9]. Aggregate bias was removed by properly specifying the regression equations [8]. A combination of aggregate and individual data has been used [18] to predict associations between particulate matter and COVID-19 mortality.

A mathematical analysis of the ecological fallacy [13] assumes a fixed set of scores that generate different linear regression coefficients or correlation values depending on whether the individual scores are used or whether they are averaged first. However, the scores may not necessarily represent an entire population. In many situations, the individual scores are themselves a sample. In such situations, the computed values of the correlation and regression coefficients of the sample of individuals are estimates of the population coefficients. The accuracy of the estimates depends on the underlying sampling distribution. Analytical sampling distributions have been derived for the Pearson R coefficient [2], coefficient of determination $R^{2}$ (see [4]), and simple regression slope *b* (see [15]) when individual scores are sampled from bivariate and multivariate normal distributions. The same sampling distributions also describe group averages for normally distributed variables [5, 17]. Thus when sampling is performed from infinitely large normally distributed populations, the population estimate of *R*, $R^{2}$ , and *b* based on a random sample of individual scores will be no more accurate than a random sample of group averages if the sample size (*n*) is the same. The disadvantage of using group averages is that *n* can be greater if the individual scores had not been first averaged since the sampling distributions do become narrower and the variances smaller when *n* increases. The advantage of using group averages is that if the group size *m* is large m≥30 , the central limit theorem does not require the variables to be normally distributed.

Less is known when sampling without replacement from finite populations which is the way data is acquired in many practical situations. This article employs Monte Carlo simulations to suggest that the *R*, $R^{2}$ , and slope distributions are also similar for samples selected without replacement for both individual and group averaged data for equal sample sizes. Our observations afford another interpretation of the ecological fallacy and suggest that for samples drawn from finite populations, the correlation coefficients and linear regression values will be selected from approximately the same sampling distribution regardless of the group size that is used.

The paper is organized as follows. Section 2 introduces the notation used in the paper and describes the analytical sampling distributions of *R*, $R^{2}$ , and slope *b*. Section 3 creates distributions by sampling without replacement from a population of size *N* using Monte Carlo simulations and compares them with analytical distributions for both simple regression (Section 3.1) and multiple regression (Section 3.2) using a small (0.5 %) and large (25 %) sample percent of the population ( $S_{n}=100\,\frac{n}{N}$ ). Section 4 explores the parameter space of ρ (the population correlation coefficient), *N*, *m*, and *n* further. Section 4.1 considers simple regression correlation, Section 4.2 discusses Fisher’s approximation, and Section 4.3 considers the linear regression slope. Mixed groups are simulated in Section 4.4 and non-normal distributions are simulated in Section 4.5. Section 4.6 considers a limited set of multiple regression examples. We conclude and discuss our observations in Section 5.

## 2 Nomenclature and analytical sampling distributions

**Table 1:** Nomenclature used in manuscript.

*N*	Size of the population
*n*	Sample size
$S_{n}=100\,\frac{n}{N}$	Sample percent of population
*m*	Group size
$n_{\mathrm{total}}$	Total number of scores used in sample = *nm*
*k*	Number of independent variables
*R*	Sample correlation coefficient
$R^{2}$	Sample coefficient of determination
*b*	Sample regression slope
$E\,\left(R\right),E\,\left(R^{2}\right),E\,\left(b\right)$	Expectation of analytical sampling distributions
$\mathrm{Var}\,\left(R\right),\mathrm{Var}\,\left(R^{2}\right),\mathrm{Var}\,\left(b\right)$	Variance of analytical sampling distributions
$E\,\left(R_{\mathrm{sim}}\right),E\,\left(R_{\mathrm{sim}}^{2}\right),E\,\left(b_{\mathrm{sim}}\right)$	Expectation of simulation sampling distributions
$\mathrm{Var}\,\left(R_{\mathrm{sim}}\right),\mathrm{Var}\,\left(R_{\mathrm{sim}}^{2}\right),\mathrm{Var}\,\left(b_{\mathrm{sim}}\right)$	Variance of simulation sampling distributions
ρ	Correlation of continuous distribution
$\rho^{2}$	Coefficient of determination of continuous distribution
$\mu_{x}$ , $\mu_{y}$	Means from bivariate distribution
$\sigma_{x},\sigma_{y}$	Standard deviation from bivariate distribution
ℬ	Bivariate distribution
*B*	Beta function
F12	Generalized hypergeometric function
$D_{R}$	Percent relative difference in Pearson *R* variance, $100\,\left(\mathrm{Var}\,\left(R_{\mathrm{sim}}\right)-\mathrm{Var}\,\left(R\right)\right)/\mathrm{Var}\,\left(R\right)$
$D_{R}^{2}$	Percent relative difference in $R^{2}$ variance, $100\,\left(\mathrm{Var}\,\left(R_{\mathrm{sim}}^{2}\right)-\mathrm{Var}\,\left(R^{2}\right)\right)/\mathrm{Var}\,\left(R^{2}\right)$
$D_{b}$	Percent relative difference in slope *b* variance, $100\,\left(\mathrm{Var}\,\left(b_{\mathrm{sim}}\right)-\mathrm{Var}\,\left(b\right)\right)/\mathrm{Var}\,\left(b\right)$
$D_{z}$	Percent relative difference in Fisher variance, $100\,\left(\mathrm{Var}\,\left(R_{z}^{\mathrm{sim}}\right)-\sigma_{z}^{2}\right)/\sigma_{z}^{2}$

We refer the reader to Table 1 which lists the symbols and their descriptions that are used in the manuscript. Muirhead [12] notes that samples $\left\{\left(x_{i},y_{i}\right):1\leq i\leq n\right\}$ selected from a bivariate distribution,

(2.1)
$$ℬ\,\left(x,y;\rho ,\mu_{x},\mu_{y},\sigma_{x},\sigma_{y}\right)=\frac{\exp\left\{-\frac{1}{2\,\left(1-\rho^{2}\right)}\,\left[{\left(\frac{x-\mu_{x}}{\sigma_{x}}\right)}^{2}+{\left(\frac{y-\mu_{y}}{\sigma_{y}}\right)}^{2}-2\,\rho \,\frac{\left(x-\mu_{x}\right)\,\left(y-\mu_{y}\right)}{\sigma_{x}\,\sigma_{y}}\right]\right\}}{2\,\pi \,\sigma_{x}\,\sigma_{y}\,{\left(1-\rho^{2}\right)}^{\frac{1}{2}}}$$

with ρ the continuous population correlation, means $\mu_{x}$ , $\mu_{y}$ , and standard deviations $\sigma_{x}$ and $\sigma_{y}$ generate the Pearson R distribution

(2.2) f(R)=(n-2)⁢Γ⁢(n-1)2⁢π⁢Γ⁢(n-12)(1-ρ2)n-12(1-ρR)-n+32(1-R2)n2-2F12(12,12;n-12;12(1+ρR)) (|R|<1),

where Γ is the gamma function, F12 is the generalized hypergeometric function. This result was originally derived by Fisher [2]. Fisher [3] also devised a transformation

(2.3) $$R_{z}=\frac{1}{2}\,\ln\left(\frac{1+R}{1-R}\right)$$

whose distribution approaches a normal distribution

$$Z\,\left(R_{z}\right)=\frac{1}{\sigma_{z}\,\sqrt{2\,\pi }}\,\exp\left(-\frac{1}{2}\,{\left(\frac{R_{z}-\mu_{z}}{\sigma_{z}}\right)}^{2}\right)$$

as n→∞ , where

(2.4) $$\mu_{z}=\frac{1}{2}\,\ln\left(\frac{1+\rho }{1-\rho }\right),\sigma_{z}^{2}=\frac{1}{n-3}.$$

Gatignon [5] notes that averages $\left({\bar{x}}_{k},{\bar{y}}_{k}\right)$ of size *m*

$${\bar{x}}_{k}=\frac{1}{m}\,\sum_{i=1}^{m}x_{i}^{\left(k\right)},{\bar{y}}_{k}=\frac{1}{m}\,\sum_{i=1}^{m}y_{i}^{\left(k\right)},1\leq k\leq n,$$

follow the distribution

(2.5) $$ℬ\,\left({\bar{x}}_{k},{\bar{y}}_{k};\rho ,\mu_{x},\mu_{y},\frac{\sigma_{x}}{\sqrt{m}},\frac{\sigma_{y}}{\sqrt{m}}\right),$$

where $x_{i}^{\left(k\right)}$ and $y_{i}^{\left(k\right)}$ refer to the *i*’th member of the *k*’th group which are drawn from a bivariate distribution ℬ , see (2.1). While the standard deviations are different in the arguments of (2.1) and (2.5), the Pearson *R* distribution (2.2) does not depend on the standard deviations. Therefore the distribution (2.2) also applies to averages $\left({\bar{x}}_{k},{\bar{y}}_{k}\right)$ (see [17]).

The expectation E⁢(R) and variation Var⁢(R) of f⁢(R) are (see [12])

(2.6) E⁢(R)=2⁢ρn-1⁢Γ⁢(n2)Γ⁢(12⁢(n-1))⁢F12⁢(12,12;12⁢(n+1);ρ2),

(2.7) Var⁢(R)=1-n-2n-1⁢(1-ρ2)⁢F12⁢(1,1;12⁢(n+1);ρ2)-E⁢(R)2.

The Var⁢(R) decreases by approximately $\frac{1}{n-1}$ as *n* increases (see [12])

(2.8) $$\mathrm{Var}\,\left(R\right)=\frac{{\left(1-\rho^{2}\right)}^{2}}{n-1}+O\,\left(n^{-2}\right).$$

In regards to the simple regression slope *b*, samples of size *n* drawn from a bivariate distribution generate the distribution h⁢(b) (see [15])

(2.9) $$h\,\left(b\right)=\frac{{\left(1-\rho^{2}\right)}^{\frac{n-1}{2}}}{\sqrt{\pi }}\,\frac{\Gamma \,\left(\frac{n}{2}\right)}{\Gamma \,\left(\frac{n-1}{2}\right)}\,\frac{\sigma_{x}}{\sigma_{y}}\,{\left[1-\rho^{2}+{\left(\rho -\frac{\sigma_{x}}{\sigma_{y}}\,b\right)}^{2}\right]}^{-\frac{n}{2}}.$$

If group averages are used, both $\sigma_{x}$ and $\sigma_{y}$ will be reduced by the square root of the group size $\sqrt{m}$ , but the ratio $\frac{\sigma_{x}}{\sigma_{y}}$ will remain the same. Thus the distribution h⁢(b) also applies to group averages for equal group sizes. The expectation E⁢(b) and variance Var⁢(b) are (see [15])

(2.10) $$E\,\left(b\right)=\rho \,\left(\frac{\sigma_{y}}{\sigma_{x}}\right),$$

(2.11) $$\mathrm{Var}\,\left(b\right)=\frac{\sigma_{y}^{2}}{\sigma_{x}^{2}}\,\left(\frac{1-\rho^{2}}{n-3}\right).$$

If we let

$$t=\frac{\left(\frac{\sigma_{x}}{\sigma_{y}}\,b-\rho \right)\,\sqrt{\nu }}{\sqrt{1-\rho^{2}}},\nu =n-1,$$

the distribution (2.9) becomes a t-distribution with ν degrees of freedom which can be approximated by a normal distribution for large *n*.

When more than one independent variable is used, the distribution of samples of size *n* drawn from a multivariate normal distribution generate the coefficient of determination $g\,\left(R^{2}\right)$ distribution as derived by Fisher [4]

(2.12) g⁢(R2)=(1-ρ2)n-12B⁢(k2,n-k-12)⁢F12⁢(n-12,n-12;k2;ρ2⁢R2)⁢(R2)k-22⁢(1-R2)n-k-32,

where *B* is the beta function, $\rho^{2}$ is the coefficient of determination for the entire population, and *k* represents the number of independent variables. Again we note that the distribution does not depend on the standard deviations of the multivariate normal distribution. Thus the sampling distribution of group averages will be the same as individual scores. The expectation and variance of $g\,\left(R^{2}\right)$ are provided by Muirhead [12],

(2.13) E⁢(R2)=1-(n-k-1n-1)⁢(1-ρ2)⁢F12⁢(1,1;n+12;ρ2),

(2.14) Var⁢(R2)=[(n-k-1)⁢(n-k+1)(n-1)⁢(n+1)]⁢(1-ρ2)2⁢F12⁢(2,2;n+32;ρ2)-[(n-k-1n-1)⁢(1-ρ2)⁢F12⁢(1,1;n+12;ρ2)]2.

## 3 Method: Monte Carlo simulations

### 3.1 Comparing simple linear regression distributions

We begin our investigation by generating a population of scores $\left\{\left(x_{i},y_{i}\right):i=1,N\right\}$ of size *N*, where each pair $\left(x_{i},y_{i}\right)$ is generated by sampling from a bivariate distribution (2.1). We generate each pair $\left(x_{i},y_{i}\right)$ by sampling $x_{i}^{*}$ and $y_{i}^{*}$ independently from a standard normal distribution and correlating them using

(3.1) $$x_{i}=\sigma_{x}\,x_{i}^{*}+\mu_{x},y_{i}=\sigma_{y}\,\left(\rho \,x_{i}^{*}+\sqrt{1-\rho^{2}}\,y_{i}^{*}\right)+\mu_{y},i=1,N,$$

where $\left(\mu_{x},\mu_{y}\right)$ represent the means and $\left(\sigma_{x},\sigma_{y}\right)$ represent the standard deviations in the bivariate distribution (2.1). The use of (3.1) does not guarantee that $\left\{\left(x_{i},y_{i}\right):i=1,N\right\}$ will be correlated at exactly the value ρ so multiple iterations are performed until they are correlated at the value of ρ to within $1\times 10^{-4}$ . We use the computed value of ρ in equations (2.6)–(2.14) when making comparisons.

Subsequently, we randomly select *n* groups of scores of size *m*. Since the sampling is done without replacement, we require that $n_{\mathrm{total}}=n\,m\leq N$ since the elements within each group will be unique and no element within the population will be used more than once in any of the groups. We also ensure that each sample is unique so that even if the same group of *nm* elements are chosen, the group arrangement will be different in each sample. Group averages are formed from the *m* scores and the Pearson *R* coefficient and linear regression slope are computed using the *n* averages. This type of sampling is used to model practical situations in which data is acquired.

**Figure 1:**
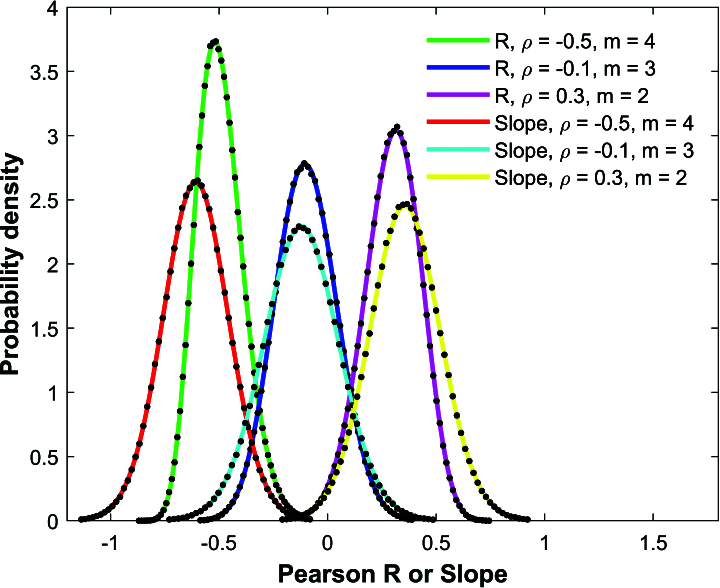
Comparison of the Monte Carlo simulation distributions (black dots) of Pearson *R* and linear regression slope using n=50 and N=10,000 with analytical distributions (2.2) in green, blue, and magenta and (2.9) in red, cyan, and yellow for ρ=-0.5,-0.1 , and 0.3 with respective group sizes m=4 , m=3 , and m=2 .

Figure 1 shows the results of the Monte Carlo simulations of the Pearson *R* correlation coefficient and linear regression slope using a population of N=10,000 scores, a sample size of n=50 , and three different values of ρ=-0.5,-0.1,0.3 each with their respective group size m=4 , m=3 , and m=2 . The population of N=10,000 scores was generated with $\left(\mu_{x},\mu_{y}\right)=\left(0,0\right)$ and $\left(\sigma_{x},\sigma_{y}\right)=\left(1,1.2\right)$ using equations (3.1). The analytical distributions for the Pearson *R* distribution (2.2) and the linear regression slope (2.9) are shown with solid lines and the simulations are shown using black dots. Each of the three simulations used a million randomly chosen samples to create the distribution. The Monte Carlo simulations visually match the analytical distributions well. However, we note that in these simulations, the sample size n=50 is small compared to the population size N=10,000 . A sample size of 50 constitutes only an $S_{n}=100\,\frac{n}{N}=0.5\%$ sample percent of the population.

**Figure 2:**
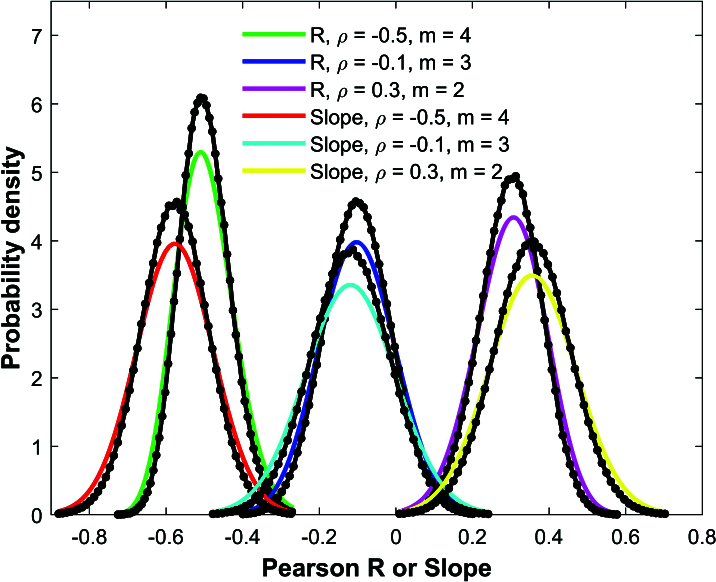
Comparison of Monte Carlo simulation distributions (black dots and lines) of Pearson *R* and linear regression slope using n=100 and N=400 with analytical distributions (2.2) in green, blue, and magenta and (2.9) in red, cyan, and yellow for ρ=-0.5,-0.1 , and 0.3 with respective group sizes m=4 , m=3 , and m=2 .

Figure 2 shows the results of the Monte Carlo simulations of the Pearson *R* correlation coefficient and linear regression slope conducted under the same conditions as Figure 1 except that a population of N=400 scores and a sample size of n=100 are used. The analytical distributions for the Pearson *R* distribution (2.2) and the linear regression slope (2.9) are shown with solid lines and the simulations are shown using black dots and lines. While the expectation values of the Monte Carlo simulations $E\,\left(R_{\mathrm{sim}}\right)$ and $E\,\left(b_{\mathrm{sim}}\right)$ seem to visibly match the expectation values E⁢(R) and E⁢(b) of the analytical distributions, the variances $\mathrm{Var}\,\left(R_{\mathrm{sim}}\right)$ and $\mathrm{Var}\,\left(b_{\mathrm{sim}}\right)$ of the simulations are visibly smaller than the analytical variances Var⁢(R) and Var⁢(b) . Note that in these simulations, the sample size n=100 represents $S_{n}=100\,\frac{n}{N}=25\%$ of the population of N=400 .

**Table 2:** Differences when comparing the analytical and simulation distribution expectation and variance in Figures 1 and 2.

Figure 1: Sample size n=50 , Population size N=10,000				
ρ	$E\,\left(R_{\mathrm{sim}}\right)-E\,\left(R\right)$	Percent relative difference in Var⁢(R)	$E\,\left(b_{\mathrm{sim}}\right)-E\,\left(b\right)$	Percent relative difference in Var⁢(b)
-0.5	$-5.9\times 10^{-5}$	-0.26	$-1.4\times 10^{-4}$	-0.56
-0.1	$-2.4\times 10^{-4}$	-0.01	$-3.2\times 10^{-4}$	-0.04
0.3	$1.7\times 10^{-4}$	-1.45	$2.8\times 10^{-4}$	-1.11
Figure 2: Sample size n=100 , Population size N=400				
ρ	$E\,\left(R_{\mathrm{sim}}\right)-E\,\left(R\right)$	Percent relative difference in Var⁢(R)	$E\,\left(b_{\mathrm{sim}}\right)-E\,\left(b\right)$	Percent relative difference in Var⁢(b)
-0.5	$4.1\times 10^{-4}$	-24.4	$7.6\times 10^{-5}$	-24.6
-0.1	$5.8\times 10^{-5}$	-23.4	$-3.5\times 10^{-4}$	-24.0
0.3	$7.1\times 10^{-5}$	-22.3	$-3.9\times 10^{-4}$	-23.2

Table 2 shows the differences in the simulated and analytical expected value of R, $E\,\left(R_{\mathrm{sim}}\right)-E\,\left(R\right)$ , the slope *b*, $E\,\left(b_{\mathrm{sim}}\right)-E\,\left(b\right)$ , and the percent relative differences in the variances

$$D_{R}=100\,\left(\frac{\mathrm{Var}\,\left(R_{\mathrm{sim}}\right)-\mathrm{Var}\,\left(R\right)}{\mathrm{Var}\,\left(R\right)}\right),D_{b}=100\,\left(\frac{\mathrm{Var}\,\left(b_{\mathrm{sim}}\right)-\mathrm{Var}\,\left(b\right)}{\mathrm{Var}\,\left(b\right)}\right)$$

for Figures 1 and 2. Table 2 shows that the differences in the expectation are small for both figures. However the percent relative differences in the variance of *R* and *b* are large in Figure 2 (ranging between -24.6% and -22.3% ). We also note that these percent relative differences are similar in magnitude to the sample percent of the population 25% in Figure 2.

### 3.2 Comparing multiple regression distributions

For multiple regression simulations, we use the Cholesky decomposition [11] to correlate the variables. If 𝐂 represents the (k+1)×(k+1) positive-definite symmetric correlation matrix, a lower triangular matrix 𝐋 is found such that $𝐂={𝐋}^{𝐓}\,𝐋$ . A vector $\vec{z}$ of k+1 variables sampled from a standard normal distribution is multiplied by $𝐋\,\vec{z}$ to form the correlated variables. In our simulations, one dependent variable *z* and two independent variables (*x* and *y*) are used ( k=2 ).

**Figure 3:**
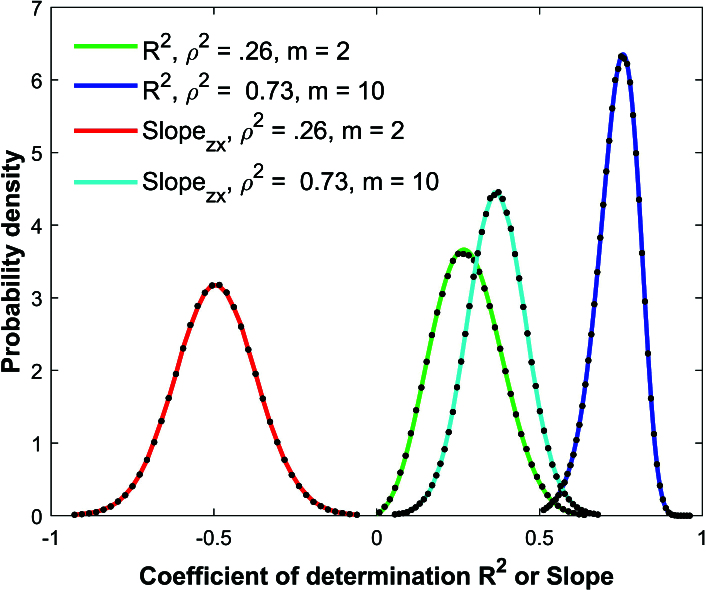
Comparison of Monte Carlo simulations (black dots) and analytical distributions of Pearson $R^{2}$ (solid green and blue) andlinear regression slope between *z* and *x* (solid red and cyan) using a sample size of n=50 and group sizes of m=2,10 in a populationof N=10,000 .

Figure 3 shows the results of the Monte Carlo simulation that generates the distribution of the coefficient of determination $R^{2}$ and the linear regression slope between *z* and *x* for a population of N=10,000 scores and a sample size of n=50 . In the first simulation, the group size is m=2 and $\rho^{2}=0.26$ . If $R_{z\,x}$ , $R_{z\,y}$ and $R_{x\,y}$ refer to the Pearson *R* correlation coefficient between *z* and *x*, *z* and *y*, and *x* and *y* respectively, the correlation matrix 𝐂 has the following off-diagonal elements: $R_{z\,x}=C_{1,2}=C_{2,1}=-0.5$ , $R_{z\,y}=C_{1,3}=C_{3,1}=0.1$ , $R_{x\,y}=C_{2,3}=C_{3,2}=-0.055$ . In the second simulation, the group size is m=10 , $\rho^{2}=0.73$ , and the correlation matrix has the following off-diagonal elements: $R_{z\,x}=C_{1,2}=C_{2,1}=0.7$ , $R_{z\,y}=C_{1,3}=C_{3,1}=0.8$ , and $R_{x\,y}=C_{2,3}=C_{3,2}=0.56$ . Each simulation shown with black dots used a million randomly chosen samples. The analytical distributions for $R^{2}$ (2.12) are shown using the green and blue solid lines. The solid red and cyan line for the linear regression slope between *z* and *x* is generated by sampling **with** replacement and is used as a proxy for the analytical distribution. This was done since an analytical distribution for multiple regression slopes could not be found in our literature search. The simulations visibly match the analytical distributions. Note that the sample size of n=50 is small compared to the population size N=10,000 and constitutes only an $S_{n}=100\,\frac{n}{N}=0.5\%$ sample percent of the population.

**Figure 4:**
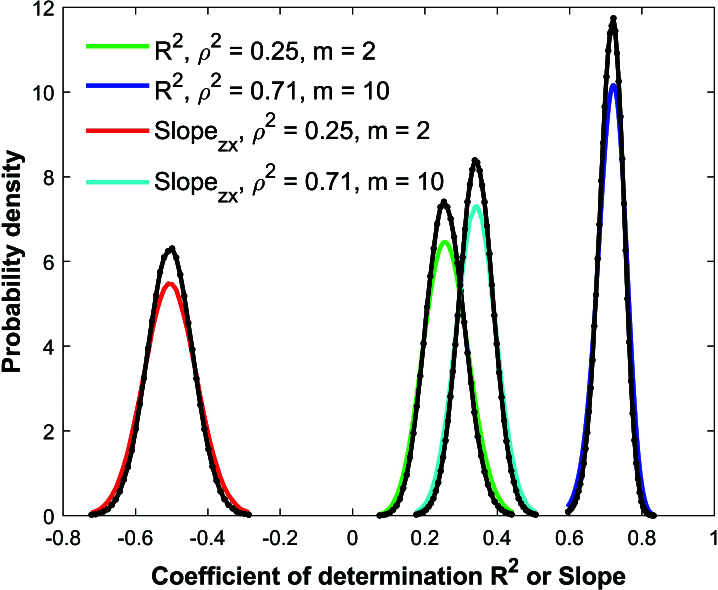
Comparison of Monte Carlo simulations (black lines and dots) and analytical distributions of Pearson $R^{2}$ (solid green and blue) and linear regression slope between *z* and *x* (solid red and cyan) using a sample size of n=150 and group sizes of m=2,10 in a population of N=600 .

Figure 4 shows the results of the Monte Carlo simulations of the coefficient of determination $R^{2}$ and the linear regression slope between *z* and *x* for a population of N=600 scores and a sample size of n=150 . In the first simulation, the group size is m=2 and $\rho^{2}=0.25$ . The correlation matrix has the following off-diagonal elements: $R_{z\,x}=C_{1,2}=C_{2,1}=-0.5$ , $R_{z\,y}=C_{1,3}=C_{3,1}=0.1$ , $R_{x\,y}=C_{2,3}=C_{3,2}=-0.12$ . In the second simulation, the group size is m=10 , $\rho^{2}=0.71$ , and the correlation matrix has the following off-diagonal elements: $R_{z\,x}=C_{1,2}=C_{2,1}=0.7$ , $R_{z\,y}=C_{1,3}=C_{3,1}=0.8$ , $R_{x\,y}=C_{2,3}=C_{3,2}=0.60$ . Each simulation shown with black lines and dots used a million randomly chosen samples. The analytical distributions for $R^{2}$ (2.12) are shown using the green and blue solid lines. The solid red and cyan line for slope between *z* and *x* is generated by sampling **with** replacement and is used as a proxy for the analytical distribution. While the expectation values of the Monte Carlo simulations $E\,\left(R_{\mathrm{sim}}^{2}\right)$ and $E\,\left(b_{\mathrm{sim}}\right)$ seem to visibly match the expectation values $E\,\left(R^{2}\right)$ and E⁢(b) of the analytical distributions, the variances $\mathrm{Var}\,\left(R_{\mathrm{sim}}^{2}\right)$ and $\mathrm{Var}\,\left(b_{\mathrm{sim}}\right)$ of the simulations are visibly smaller than the analytical variances $\mathrm{Var}\,\left(R^{2}\right)$ and Var⁢(b) . Note that in these simulations, the sample size n=150 represents $S_{n}=100\,\frac{n}{N}=25\%$ of the population of N=600 .

Table 3 shows the differences in the simulated and analytical expected value of $R^{2}$ , $E\,\left(R_{\mathrm{sim}}^{2}\right)-E\,\left(R^{2}\right)$ , the linear regression slope *b* between *z* and *x*, $E\,\left(b_{\mathrm{sim}}\right)-E\,\left(b\right)$ , and the percent relative difference in the variances

$$D_{R}^{2}=100\,\left(\frac{\mathrm{Var}\,\left(R_{\mathrm{sim}}^{2}\right)-\mathrm{Var}\,\left(R^{2}\right)}{\mathrm{Var}\,\left(R^{2}\right)}\right),D_{b}=100\,\left(\frac{\mathrm{Var}\,\left(b_{\mathrm{sim}}\right)-\mathrm{Var}\,\left(b\right)}{\mathrm{Var}\,\left(b\right)}\right)$$

for Figures 3 and 4. Table 3 shows that the differences in the expectation are small for both figures. However the percent relative difference in the variance of $R^{2}$ and *b* are large in Figure 4 (ranging between -24.7% and -23.0% ). We also note that these percent relative differences are similar in magnitude to the sample percent of the population $S_{n}=25\%$ in Figure 4.

**Table 3:** Differences when comparing the analytical and simulation distributions for Figures 3 and 4.

Figure 3: Sample size n=50 , Population size N=10000				
$\rho^{2}$	$E\,\left(R_{\mathrm{sim}}^{2}\right)-E\,\left(R^{2}\right)$	Percent relative differences in $\mathrm{Var}\,\left(R^{2}\right)$	$E\,\left(b_{\mathrm{sim}}\right)-E\,\left(b\right)$	Percent relative differences in Var⁢(b)
0.26	$-2.1\times 10^{-4}$	$1.1\times 10^{-2}$	$-5.6\times 10^{-5}$	$-5.3\times 10^{-3}$
0.73	$-7.1\times 10^{-5}$	$-7.6\times 10^{-3}$	$-2.8\times 10^{-5}$	$-5.2\times 10^{-3}$
Figure 4: Sample size n=150 , Population size N=600				
$\rho^{2}$	$E\,\left(R_{\mathrm{sim}}^{2}\right)-E\,\left(R^{2}\right)$	Percent relative differences in $\mathrm{Var}\,\left(R^{2}\right)$	$E\,\left(b_{\mathrm{sim}}\right)-E\,\left(b\right)$	Percent relative differences in Var⁢(b)
0.25	$-1.7\times 10^{-3}$	-23.0	$-1.8\times 10^{-4}$	-24.3
0.71	$-3.5\times 10^{-4}$	-24.7	$4.2\times 10^{-7}$	-23.5

These first examples suggest that when sampling without replacement from normal distributions, the expectation differences between the analytical and simulated distributions are small regardless of the group size *m*. Differences arise between the variance of the analytical and simulated distributions when the sample size *n* is a significant percent of the population size *N* regardless of the group size *m*. In the next section, we explore parameter space further to determine if the differences in the analytical and simulated expectations in *R*, $R^{2}$ and the linear regression slope remain small. We also investigate if the differences in variances are a function of the sample percent of the population $S_{n}=100\,\frac{n}{N}$ .

## 4 Results: Exploring parameter space

Our objective in Section 4 is to continue to explore the parameter space of ρ, *m*, *n*, and *N* further to determine if and where differences exist between the analytical distributions and the sampling distributions created without replacement. The parallel Fortran code is available at: https://github.com/davytorres/MonteCarloEcological.git. In our preliminary analysis in Section 3, only one population was chosen for the simulations. We increase the number of populations to 112 (chosen because it is divisible by 8 and 14 which are the number of processors available on our computers for parallel runs) to determine if the population selection affects the observed trends. In all the simulations in Section 4, a million randomly chosen samples are used to create each distribution. The most important parameter we identified in Section 3 was the sample percent of the total population $S_{n}=100\,\frac{n}{N}$ which affects the variances of the distributions. Other parameters such as the group size and the Pearson ρ value did not seem to have a significant impact when comparing the analytical and simulated distributions. We also explore mixed groups sizes and non-normal distributions.

### 4.1 Simple regression – Pearson R

**Figure 5:**
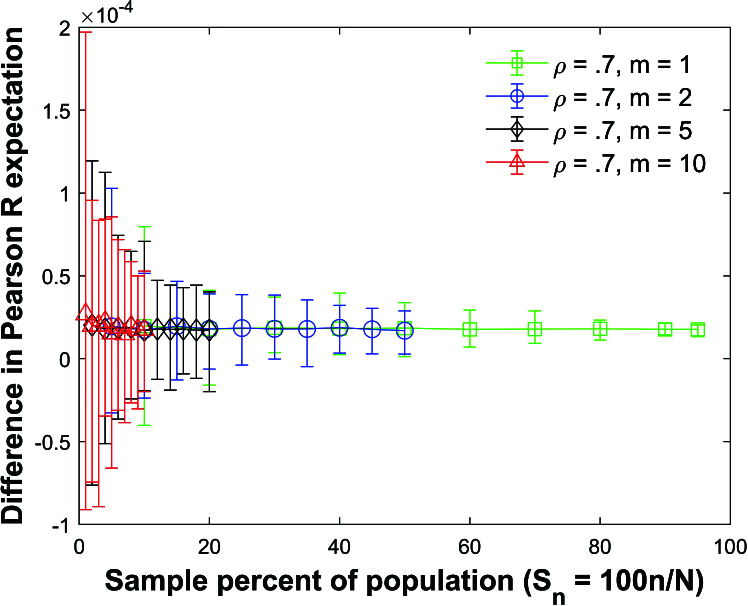
Plot of difference $\left(E\,\left(R_{\mathrm{sim}}\right)-E\,\left(R\right)\right)$ using a population size N=10,000 sampled from a bivariate distribution with ρ=0.7 plotted against the sample percent of the population $S_{n}=100\,\frac{n}{N}$ . The plot symbol shows the average difference and the error bars show the maximum and minimum differences using 112 different populations of size N=10,000 .

Figure 5 plots the difference $\left(E\,\left(R_{\mathrm{sim}}\right)-E\,\left(R\right)\right)$ using a population size N=10,000 sampled from a bivariate distribution with ρ=0.7 , $\left(\mu_{x},\mu_{y}\right)=\left(0,0\right)$ , and $\left(\sigma_{x},\sigma_{y}\right)=\left(1,1\right)$ ; E⁢(R) is computed using equation (2.6); $E\,\left(R_{\mathrm{sim}}\right)$ is generated with Monte Carlo simulations that use different sample sizes *n* (displayed along the horizontal axis using the sample percent of the population $S_{n}=100\,\frac{n}{N}$ ) and different group sizes m=1,2,5,10 . The symbol on the plot (square, circle, diamond, or triangle) shows the average difference and the error bars show the maximum and minimum differences from 112 different populations with size N=10,000 . Due to the number of computations required, simulations were run in parallel on fourteen processors on a laptop or eight processors on a desktop using MPI Fortran. Note that the sample percent of the population is limited according to the equation n⁢m≤N . For example, a group size of m=2 can only use 50% of the population. For this reason, the values we use for $S_{n}$ along the horizontal axis for each group size vary according to $S_{n}=10\,\frac{i}{m}$ , i=1,10 . The exception is m=1 . We modify the largest value of $S_{n}$ when m=1 and i=10 to be 95 since only one sample can be chosen when m=1 and all the elements of the population are used.

Figure 5 shows that the difference in $\left(E\,\left(R_{\mathrm{sim}}\right)-E\,\left(R\right)\right)$ is small ( $<2\times 10^{-4}$ ) confirming that the expectation values can be approximated using (2.6) regardless of the sample size *n* and group size *m* for normally distributed variables and ρ=0.7 . There is a small positive offset in the average difference. Simulations (figure not shown to save space) identical to Figure 5 were also conducted except that a small population N=400 was used. In these simulations, all the absolute average differences were small ( $<5\times 10^{-4}$ ) but larger than the average differences shown in Figure 5.

**Figure 6:**
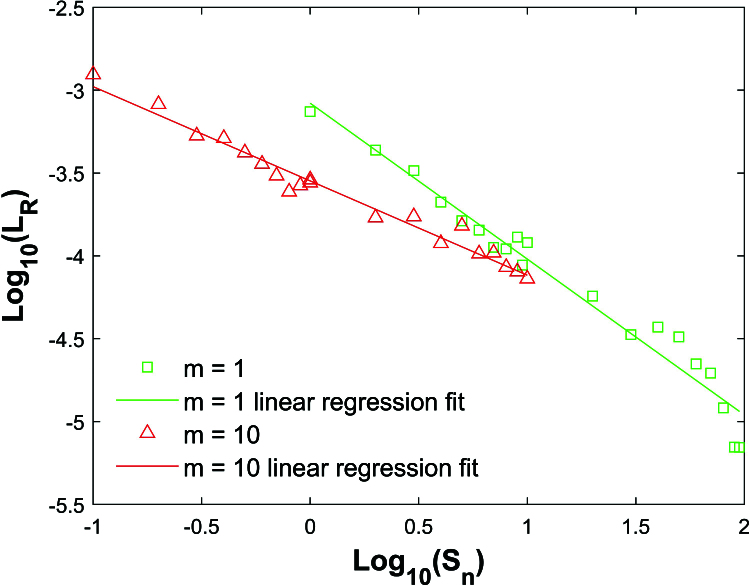
Plot of ${\mathrm{Log}}_{10}\left(L_{R}\right)$ vs ${\mathrm{Log}}_{10}\left(S_{n}\right)$ using a population size N=10,000 sampled from a bivariate distribution with ρ=0.7 for m=1 and m=10 . The slopes of the linear regression lines are: -0.94 (m=1),-0.71 (m=2),-0.61 (m=5),-0.57 (m=10) . The plots for m=2 and m=5 are not shown to avoid a cluttered plot.

We also note that the range of the error bars in Figure 5 increases as the sample percent of the population decreases. Define the range $L_{R}$ of the error bars using (4.1),

(4.1) $$L_{R}=\mathrm{Max}\left\{E\,\left(R_{\mathrm{sim}}\right)-E\,\left(R\right)\right\}-\mathrm{Min}\left\{E\,\left(R_{\mathrm{sim}}\right)-E\,\left(R\right)\right\}.$$

If the ${\mathrm{Log}}_{10}\left(L_{R}\right)$ is plotted against ${\mathrm{Log}}_{10}\left(S_{n}\right)$ , a linear regression line can be fit through the data as shown in Figure 6 for m=1 and m=10 . To avoid an overly cluttered plot, the plots for m=2 and m=5 are not shown. Additional points for small values of $S_{n}$ are included in the plot $\left\{S_{n}=\frac{i}{m}:i=1,10\right\}$ in addition to the points $\left\{S_{n}=10\,\frac{i}{m}:i=1,10\right\}$ included in Figure 5. Based on the slope of linear regression fit, the range of the error bars $L_{R}$ varies according to $S_{n}^{\alpha }$ , where α=-0.94 (m=1) , α=-0.71 (m=2) , α=-0.61 (m=5) , and α=-0.57 (m=10) in their respective ranges of $S_{n}$ .

**Figure 7:**
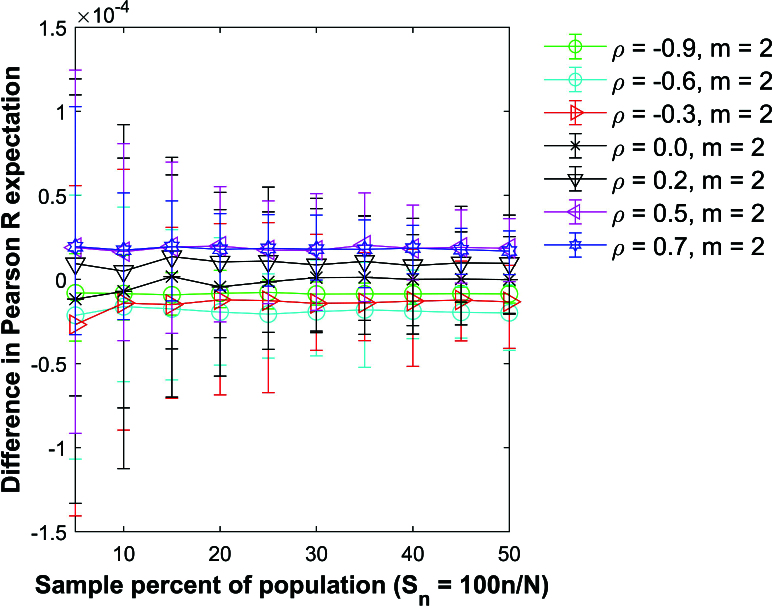
Plot of difference $\left(E\,\left(R_{\mathrm{sim}}\right)-E\,\left(R\right)\right)$ using a population size N=10,000 sampled from a bivariate distribution with different values of ρ with group size m=2 plotted against the sample percent of the population $S_{n}=100\,\frac{n}{N}$ . The plot symbol shows the average difference and the error bars show the maximum and minimum differences using 112 different populations of size N=10,000 .

**Figure 8:**
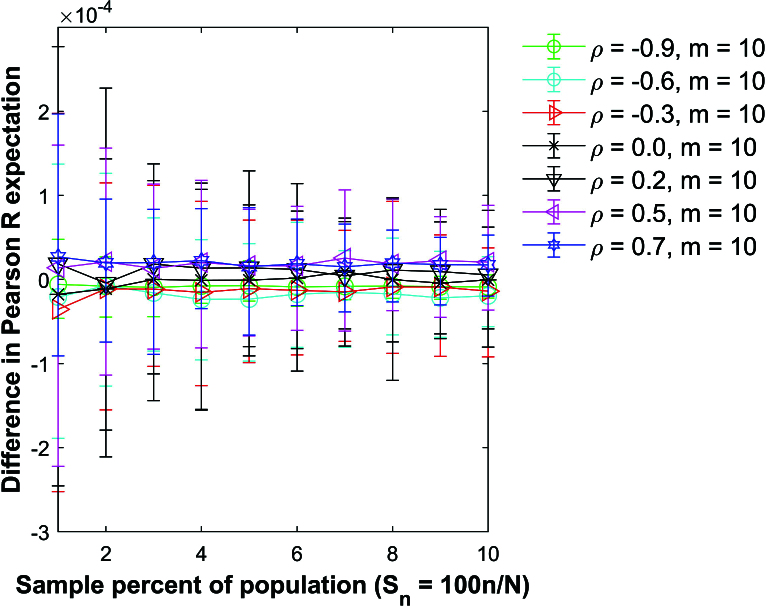
Plot of difference $\left(E\,\left(R_{\mathrm{sim}}\right)-E\,\left(R\right)\right)$ using a population size N=10,000 sampled from a bivariate distribution with different values of ρ with group size m=10 plotted against the sample percent of the population $S_{n}=100\,\frac{n}{N}$ . The plot symbol shows the average difference and the error bars show the maximum and minimum differences using 112 different populations of size N=10,000 .

Figure 7 plots the difference $\left(E\,\left(R_{\mathrm{sim}}\right)-E\,\left(R\right)\right)$ using a population size N=10,000 sampled from a bivariate distribution with different values of ρ={-0.9,-0.6,-0.3,0.0,0.2,0.5,0.7} and group size m=2 . Figure 8 shows the results for m=10 . Except for the value of ρ, the Monte Carlo simulation is conducted under the same conditions described in Figure 5. The plot symbol shows the average difference and the error bars show the maximum and minimum differences using 112 different populations. The differences remain small regardless of the value of ρ used. The expectation $E\,\left(R_{\mathrm{sim}}\right)$ is slightly larger than E⁢(R) for ρ>0 , and $E\,\left(R_{\mathrm{sim}}\right)$ is slightly less than E⁢(R) for ρ<0 .

**Figure 9:**
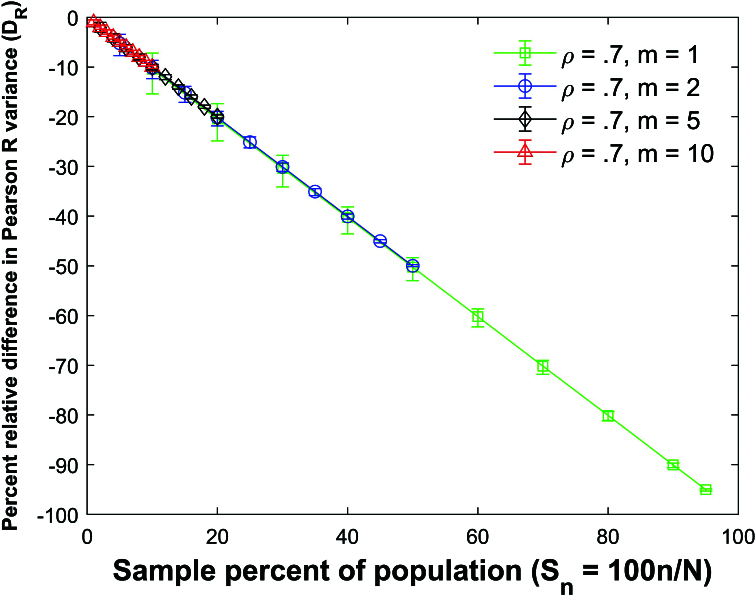
Plot of percent relative difference $D_{R}=100\,\left(\frac{\mathrm{Var}\,\left(R_{\mathrm{sim}}\right)-\mathrm{Var}\,\left(R\right)}{\mathrm{Var}\,\left(R\right)}\right)$ using a population size N=10,000 sampled from a bivariate distribution with ρ=0.7 plotted against the sample percent of the population $S_{n}=100\,\frac{n}{N}$ . The plot symbol shows the average difference and the error bars show the maximum and minimum differences using 112 different populations. The percent relative difference can be described by the linear equation $D_{R}=-S_{n}$ . The range of the error bars increases as the sample percent of the population decreases and the group size decreases.

Figure 9 plots the percent relative difference $D_{R}=100\,\left(\frac{\mathrm{Var}\,\left(R_{\mathrm{sim}}\right)-\mathrm{Var}\,\left(R\right)}{\mathrm{Var}\,\left(R\right)}\right)$ using a population size N=10,000 sampled from a bivariate distribution with ρ=0.7 . The plot symbol (square, circle, diamond, or triangle) shows the average difference and the error bars show the maximum and minimum differences using 112 different populations. The variance Var⁢(R) is computed using (2.7) and $\mathrm{Var}\,\left(R_{\mathrm{sim}}\right)$ is generated using a Monte Carlo simulation in which a million samples were selected without replacement using different sample sizes *n* (displayed along the horizontal axis using the sample percent of the population $S_{n}=100\,\frac{n}{N}$ ).

The average percent relative differences can be closely approximated by the linear plot

(4.2) $$D_{R}=-S_{n}$$

which is **not a function of the group size *m***. The size of the error bars increases as the sample percent of the population decreases which was also observed in the Pearson *R* expectation plot (Figure 5).

It is well known if all samples are considered, the variance of the sampling distribution $\sigma_{m}^{2}$ is reduced by the group size *m* when sampling with replacement

$$\sigma_{m}^{2}=\frac{\sigma^{2}}{m}.$$

When computing the variance when sampling without replacement ${\left(\sigma_{m}^{2}\right)}_{w\,r}$ , there is an additional finite population correction $\frac{N-m}{N-1}$ ,

$${\left(\sigma_{m}^{2}\right)}_{w\,r}=\frac{\sigma^{2}}{m}\,\left(\frac{N-m}{N-1}\right).$$

The percent relative difference between the two variances is

(4.3) $$100\,\left(\frac{{\left(\sigma_{m}^{2}\right)}_{w\,r}-\sigma_{m}^{2}}{\sigma_{m}^{2}}\right)=-100\,\left(\frac{m-1}{N-1}\right)$$

which is similar to the approximation (4.2) $D_{R}=-100\,\frac{n}{N}=-S_{n}$ (when *n* is substituted for m-1 and N-1 replaced with *N*). However, (4.3) does not apply to the Pearson *R* coefficient and linear regression slope since the variance is computed from the Pearson *R* coefficient and slope after they are calculated from each sample composed of *n* groups each of size *m*.

**Figure 10:**
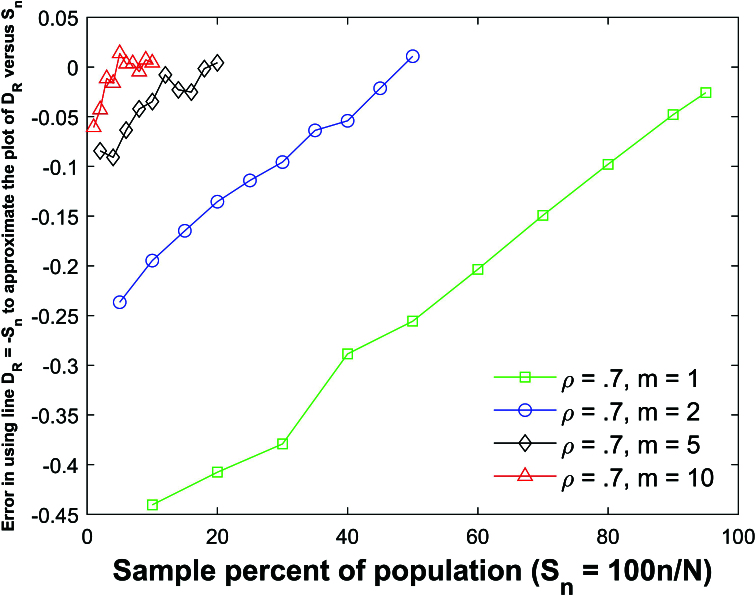
Plot of error $D_{R}+S_{n}$ (from Figure 9) when approximating the plot of $D_{R}$ versus $S_{n}$ with the line $D_{R}=-S_{n}$ . The average value of the $D_{R}$ from the 112 populations is used in the plot. The absolute error increases when the group size decreases and the sample percent of the population $S_{n}$ decreases.

Figure 10 plots the error (from Figure 9) when approximating the plot of $D_{R}$ versus $S_{n}$ with the line $D_{R}=-S_{n}$ . The error plotted is $D_{R}+S_{n}$ . All absolute errors are less than 0.5 %. We note that the absolute error increases when the group size decreases and the sample percent of the population $S_{n}$ decreases.

Simulations (not shown) identical to Figure 10 were conducted except that a small population N=400 was used. In these simulations, all the absolute average errors were small ( <1.2% ) but larger than the average errors shown in Figure 10.

To further test the validity of (4.2), we fit a linear regression line through the plot of $D_{R}$ versus $S_{n}$ shown in Figure 9 for different group sizes m=1,2,5,10 and different values of ρ=-0.9,-0.6,-0.3,0.0,0.2,0.5 , and 0.7. The linear regression line fit through all values of *m* and all values of ρ has a Pearson *R* value in the range $-1\leq R<-1+\left(2\times 10^{-5}\right)$ , a slope *b* in the range $-1-\left(1\times 10^{-3}\right)<b<-1+(7\times 10^{-3}$ ), and a y-intercept $y_{\mathrm{int}}$ in the range $-6\times 10^{-3}<y_{\mathrm{int}}<1\times 10^{-3}$ which shows that (4.2) is a good approximation to the plot of $D_{R}$ vs $S_{n}$ .

The ecological fallacy can be viewed from the following perspective for normally distributed variables and random sampling. Note that the total number of scores $n_{\mathrm{total}}$ used for each $S_{n}$ is *mn* since *n* groups of *m* scores are averaged before the Pearson *R* coefficient is computed. For a fixed $n_{\mathrm{total}}$ , smaller group sizes (*m*) have the advantage of allowing larger sample sizes as $n=\frac{n_{\mathrm{total}}}{m}$ . The variance of the sampling distribution decreases as the sample size (*n*) increases for two reasons. First Var⁢(R) scales as $\frac{1}{n-1}$ according to (2.8). Secondly, when sampling without replacement, the variance is further reduced according to the $D_{R}=-S_{n}$ from Figure 9. Thus for a fixed set of available scores $n_{\mathrm{total}}$ , smaller group sizes translate into larger sample sizes and smaller variances in the sampling distributions. Smaller variances mean the sample correlation has a higher probability of being close to the population correlation.

### 4.2 Testing Fisher’s approximation

Since the distribution of *R* is not symmetric about the mean, we investigated properties of the Fisher transformed (2.3) values of *R* as the transformed distribution is approximately normal and is useful in building confidence intervals. Recall from (2.4) that as n→∞ , the Fisher transformed variables $R_{z}=\frac{1}{2}\,\ln\left(\frac{1+R}{1-R}\right)$ approach a normal distribution with mean

$$\mu_{z}=\frac{1}{2}\,\ln\left(\frac{1+\rho }{1-\rho }\right)$$

and variance

$$\sigma_{z}^{2}=\frac{1}{n-3}.$$

Figures 11–13 use the same data and parameters from the simulations described in Figures 5–10.

**Figure 11:**
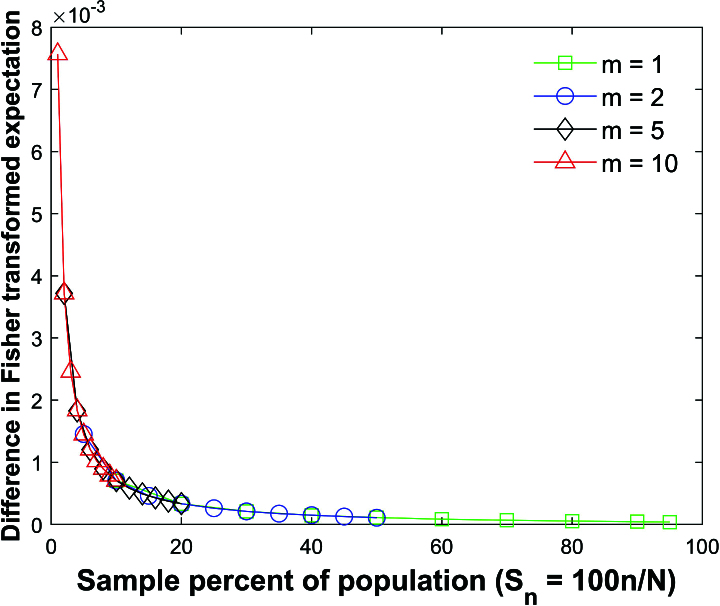
Plot of difference $\left(E\,\left(R_{z}^{\mathrm{sim}}\right)-\mu_{z}\right)$ , where ρ=0.7 , $\mu_{z}=\frac{1}{2}\,\ln\left(\frac{1+\rho }{1-\rho }\right)$ , and $E\,\left(R_{z}^{\mathrm{sim}}\right)$ is the expectation of the distribution formedby the Monte Carlo simulation after applying the transformation $R_{z}=\frac{1}{2}\,\ln\left(\frac{1+R}{1-R}\right)$ .

Figure 11 plots the average difference $\left(E\,\left(R_{z}^{\mathrm{sim}}\right)-\mu_{z}\right)$ over the 112 populations where $E\,\left(R_{z}^{\mathrm{sim}}\right)$ is the expectation of the distribution formed by the transformed

$$R_{z}=\frac{1}{2}\,\ln\left(\frac{1+R}{1-R}\right)$$

variables created by Monte Carlo simulations with ρ=0.7 . We note that the difference is small but increases as $S_{n}$ decreases. A plot of ${\mathrm{Log}}_{10}\left(E\,\left(R_{z}^{\mathrm{sim}}\right)-\mu_{z}\right)$ vs ${\mathrm{Log}}_{10}\left(S_{n}\right)$ is linear and the least squares slope of the line is -1.1 with a Pearson R value of -0.997, from which one can conclude that $\left(E\,\left(R_{z}^{\mathrm{sim}}\right)-\mu_{z}\right)$ decreases as $S_{n}^{\alpha }$ , where α=-1.1 for the ranges of $S_{n}$ considered.

**Figure 12:**
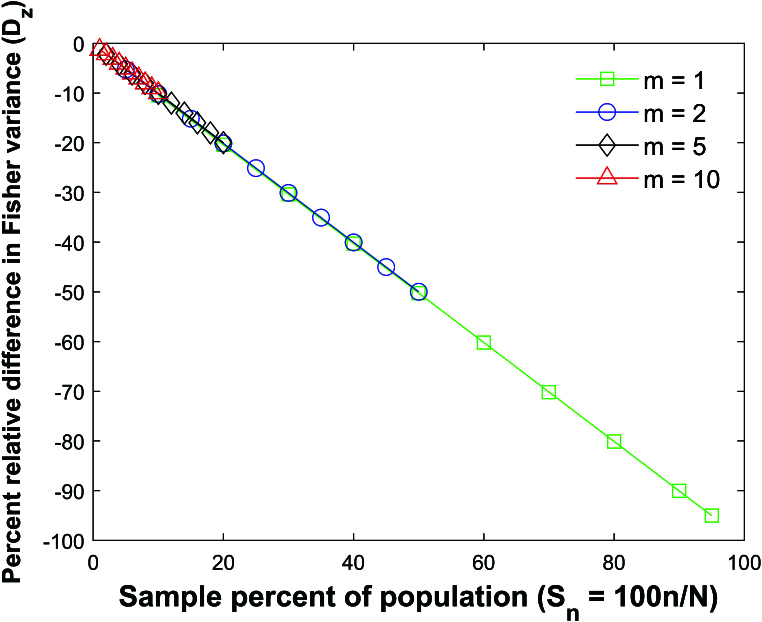
Plot of percent relative difference $100\,\left(n-3\right)\,\left(\mathrm{Var}\,\left(R_{z}\right)-\frac{1}{n-3}\right)$ , where $\mathrm{Var}\,\left(R_{z}^{\mathrm{sim}}\right)$ is the variance of the distribution formedby the Monte Carlo simulation after applying the transformation $R_{z}=\frac{1}{2}\,\ln\left(\frac{1+R}{1-R}\right)$ with ρ=0.7 .

Figure 12 plots the average percent relative difference in the variance

$$D_{z}=100\,\left(\frac{\mathrm{Var}\,\left(R_{z}^{\mathrm{sim}}\right)-\sigma_{z}^{2}}{\sigma_{z}^{2}}\right)$$

$$=100\,\left(n-3\right)\,\left(\mathrm{Var}\,\left(R_{z}^{\mathrm{sim}}\right)-\frac{1}{n-3}\right),$$

where $\mathrm{Var}\,\left(R_{z}^{\mathrm{sim}}\right)$ is the variance of the distribution formed by the Monte Carlo simulation after applying the Fisher transformation. Note that $D_{z}$ can be approximated with the line $D_{z}=-S_{n}$ . The absolute errors in approximating the plot of $D_{z}$ vs $S_{n}$ with the line $D_{z}=-S_{n}$ are all less than 0.5 percent. As with $D_{R}$ , the absolute error increases when the group size decreases and the sample percent of the population $S_{n}$ decreases.

**Figure 13:**
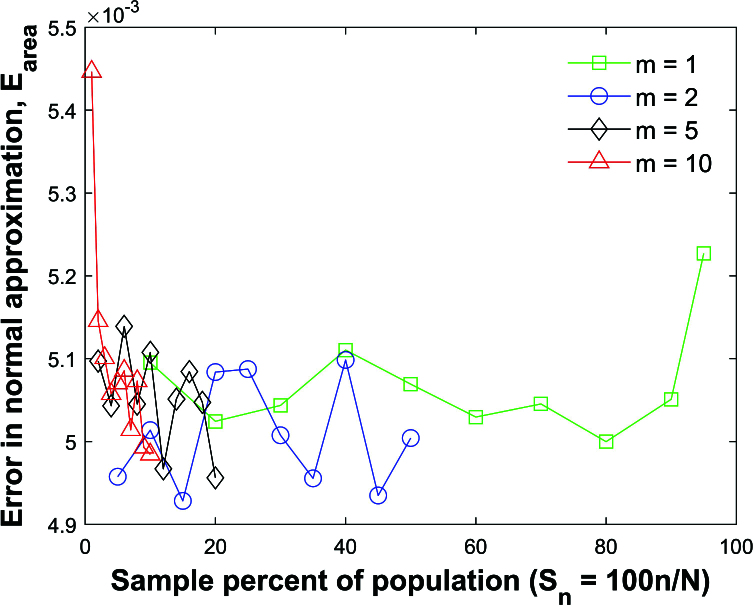
Plot of error $E_{\mathrm{area}}=\int_{-3.5}^{3.5}\left|f_{\mathrm{sim}}\,\left(R_{z}\right)-Z\,\left(R_{z}\right)\right|\,𝑑z$ , where $Z\,\left(R_{z}\right)=\frac{1}{\sigma \,\sqrt{2\,\pi }}\,\exp\left(-\frac{1}{2}\,{\left(\frac{R_{z}-\mu }{\sigma }\right)}^{2}\right)$ and $f_{\mathrm{sim}}\,\left(R_{z}\right)$ is the distributionformed by the Monte Carlo simulation after applying the transformation $R_{z}=\frac{1}{2}\,\ln\left(\frac{1+R}{1-R}\right)$ .

We have compared the expectation and variances of the Pearson *R* distribution. To compare the simulated and analytical distribution functions, we use the Fisher transformed variables. Figure 13 plots the average error

$$E_{\mathrm{area}}=\int_{-3.5}^{3.5}\left|f_{\mathrm{sim}}\,\left(R_{z}\right)-Z\,\left(R_{z}\right)\right|\,𝑑z,$$

where

$$Z\,\left(R_{z}\right)=\frac{1}{\sigma \,\sqrt{2\,\pi }}\,\exp\left(-\frac{1}{2}\,{\left(\frac{R_{z}-\mu }{\sigma }\right)}^{2}\right)$$

and $f_{\mathrm{sim}}\,\left(R_{z}\right)$ is the distribution formed by the Monte Carlo simulation. Since the variance decreases with $S_{n}$ when sampling without replacement, the mean μ and standard deviation σ were selected to be the mean and standard deviation of the simulated distribution so Figure 13 is essentially a test of normality; $E_{\mathrm{area}}$ is small ( $<5.5\times 10^{-3}$ ) for the tested range of $S_{n}$ for all group sizes *m*.

### 4.3 Simple regression – Slope

**Figure 14:**
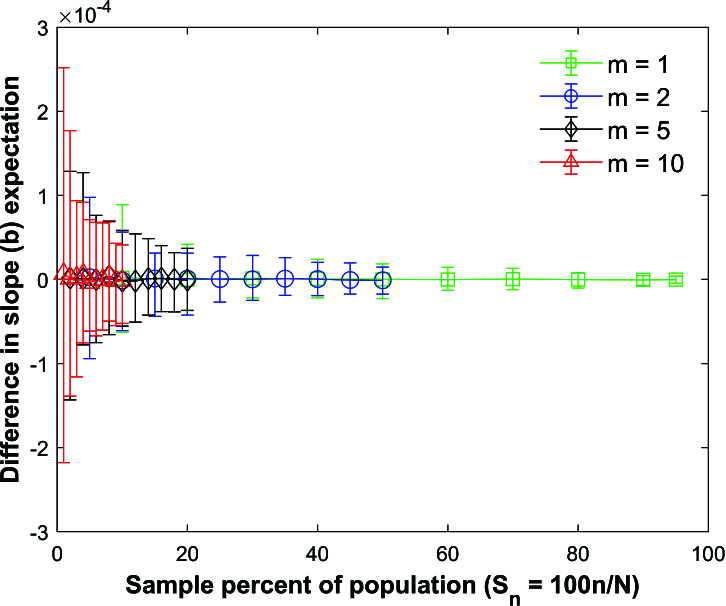
Plot of difference $\left(E\,\left(b_{\mathrm{sim}}\right)-E\,\left(b\right)\right)$ using a population size N=10,000 sampled from a bivariate distribution with ρ=0.7 .The plot symbol shows the average difference and the error bars show the maximum and minimum differences using 112 different populations.

**Figure 15:**
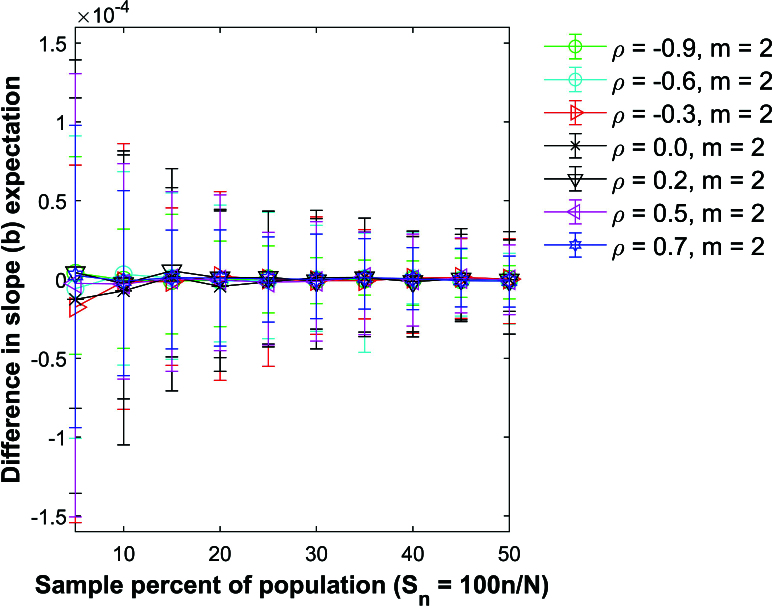
Plot of difference $\left(E\,\left(b_{\mathrm{sim}}\right)-E\,\left(b\right)\right)$ using a population size N=10,000 sampled from a bivariate distribution with different values of ρ with group size m=2 plotted against the sample percent of the population $S_{n}$ .

**Figure 16:**
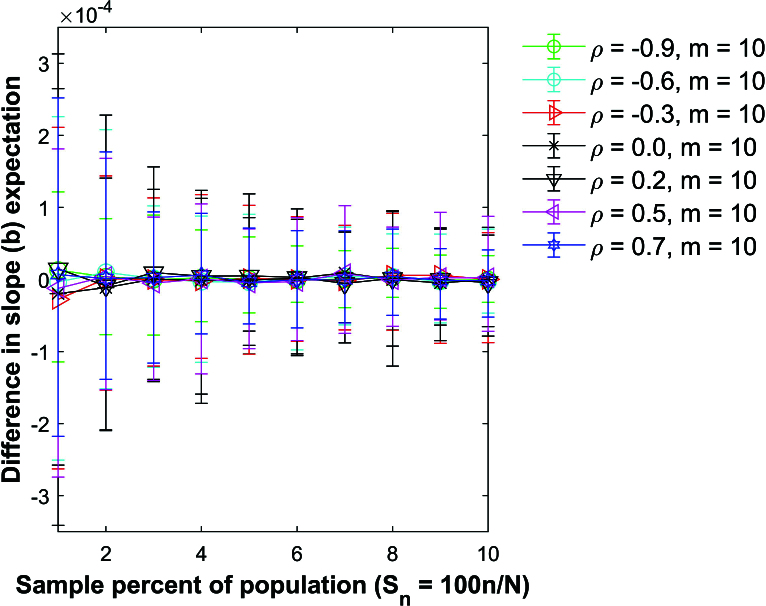
Plot of difference $\left(E\,\left(b_{\mathrm{sim}}\right)-E\,\left(b\right)\right)$ using a population size N=10,000 sampled from a bivariate distribution with different values of ρ with group size m=10 plotted against the sample percent of the population $S_{n}$ .

**Figure 17:**
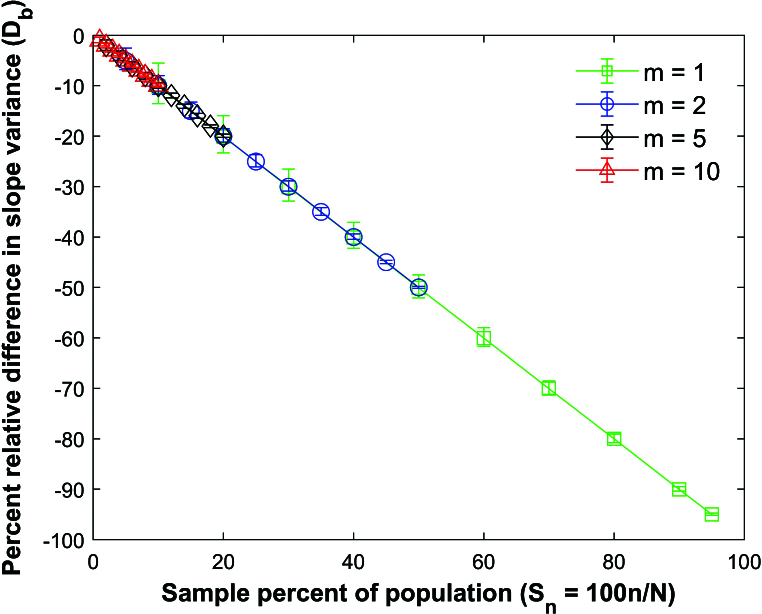
Plot of percent relative difference $D_{b}=100\,\left(\frac{\mathrm{Var}\,\left(b_{\mathrm{sim}}\right)-\mathrm{Var}\,\left(b\right)}{\mathrm{Var}\,\left(b\right)}\right)$ using a population size N=10,000 sampled from a bivariate distribution with ρ=0.7 . The percent relative difference can be described by the linear plot $D_{b}=-S_{n}$ . The size of the error bars increases as the sample percent of the population decreases and the group size decreases.

**Figure 18:**
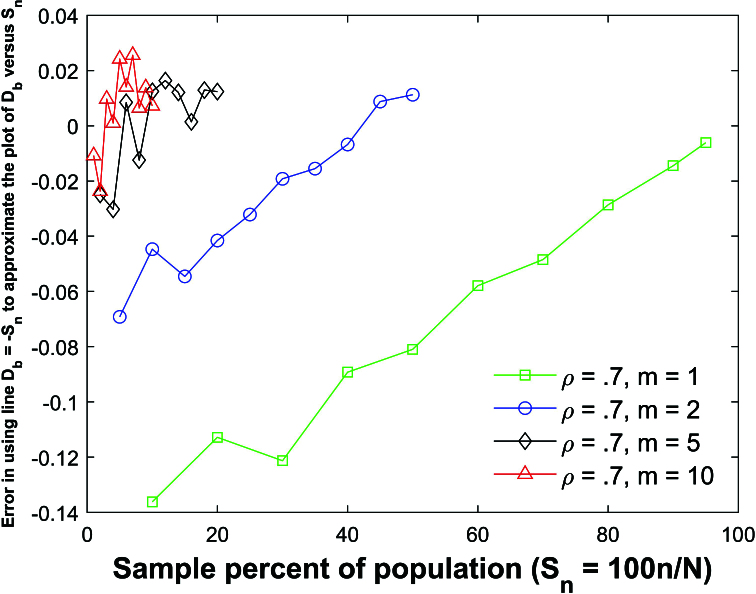
Plot of error $D_{b}+S_{n}$ (from Figure 17) when approximating the plot of $D_{b}$ versus $S_{n}$ with the line $D_{b}=-S_{n}$ . The absolute value of the error increases when the group size decreases and the sample percent of the population $S_{n}$ decreases (for m=1 and m=2 ).

Figures 14–18 use the same data from the simulations described in Figures 5–10. Figure 14 plots the difference $\left(E\,\left(b_{\mathrm{sim}}\right)-E\,\left(b\right)\right)$ . The difference $\left(E\,\left(b_{\mathrm{sim}}\right)-E\,\left(b\right)\right)$ is small confirming that the expectation values can be approximated using (2.10) regardless of the sample size *n* and group size *m* for normally distributed variables and ρ=0.7 . Simulations identical to Figure 14 were also conducted except that a small population N=400 was used. In these simulations, all the absolute average differences were small ( $<2\times 10^{-4}$ ) but larger than the average differences shown in Figure 14.

Similar to Figure 5, the range of the error bars in Figure 14 increases as the sample percent of the population decreases. Define the range $L_{b}$ of the error bars using (4.4),

(4.4) $$L_{b}=\mathrm{Max}\left\{E\,\left(b_{\mathrm{sim}}\right)-E\,\left(b\right)\right\}-\mathrm{Min}\left\{E\,\left(b_{\mathrm{sim}}\right)-E\,\left(b\right)\right\}.$$

If the ${\mathrm{Log}}_{10}\left(L_{b}\right)$ is plotted against ${\mathrm{Log}}_{10}\left(S_{n}\right)$ , a linear regression line can be fit through the data. Additional points for small values of $S_{n}$ are included in the plot $\left\{S_{n}=\frac{i}{m}:i=1,10\right\}$ in addition to the points $\left\{S_{n}=10\,\frac{i}{m}:i=1,10\right\}$ . Based on the slope of linear regression fit, the range of the error bars $L_{b}$ varies according to $S_{n}^{\alpha }$ , where α=-0.92 (m=1) , α=-0.72 (m=2) , α=-0.60 (m=5) , α=-0.58 (m=10) for their respective ranges of $S_{n}$ .

Figure 15 plots the difference $\left(E\,\left(b_{\mathrm{sim}}\right)-E\,\left(b\right)\right)$ using a population size N=10,000 sampled from a bivariate distribution with different values of ρ={-0.9,-0.6,-0.3,0.0,0.2,0.5,0.7} and group size m=2 . Figure 16 shows the results for m=10 . The differences remain small regardless of the value of ρ used.

Figure 17 plots the percent relative difference

$$D_{b}=100\,\left(\frac{\mathrm{Var}\,\left(b_{\mathrm{sim}}\right)-\mathrm{Var}\,\left(b\right)}{\mathrm{Var}\,\left(b\right)}\right)$$

using a population size N=10,000 sampled from a bivariate distribution with ρ=0.7 . Var⁢(b) is computed using (2.11) and $\mathrm{Var}\,\left(b_{\mathrm{sim}}\right)$ is generated using a Monte Carlo simulation in which a million samples were selected without replacement. The average percent relative difference can be described by the linear plot

(4.5) $$D_{b}=-S_{n},$$

where $S_{n}=100\,\frac{n}{N}$ which is **not a function of the group size m **. However, the size of the error bars increases as the sample percent of the population decreases and the group size decreases. Figure 18 plots the error (from Figure 17) when approximating the plot of $D_{b}$ versus $S_{n}$ with the line $D_{b}=-S_{n}$ . The error plotted is $D_{b}+S_{n}$ . All absolute average errors are less than 0.14 %. We note that the absolute value of the error increases when the group size decreases and the sample percent of the population $S_{n}$ decreases (for m=1 and m=2 ).

Simulations (not shown) identical to Figure 18 were conducted except that a small population N=400 was used. In these simulations, all the absolute average errors were small (0.6 %) but larger than the average errors shown in Figure 18.

To test the validity of (4.5), we fit a linear regression line through the plot of $D_{b}$ versus $S_{n}$ shown in Figure 17 for different group sizes m=1,2,5,10 and different values of ρ=-0.9,-0.6,-0.3,0.0,0.2,0.5 , and 0.7. The linear regression line fit through all values of *m* and all values of ρ has a Pearson *R* value in the range $-1\leq R<-1+\left(1.8\times 10^{-5}\right)$ , a slope *b* in the range $-1+\left(-4\times 10^{-3}\right)<b<-1+\left(4\times 10^{-3}\right)$ , and a y-intercept $y_{\mathrm{int}}$ in the range $-3\times 10^{-3}<y_{\mathrm{int}}<3\times 10^{-3}$ which shows that (4.5) is a good approximation to the plot of $D_{b}$ vs $S_{n}$ .

### 4.4 Groups of mixed size

**Figure 19:**
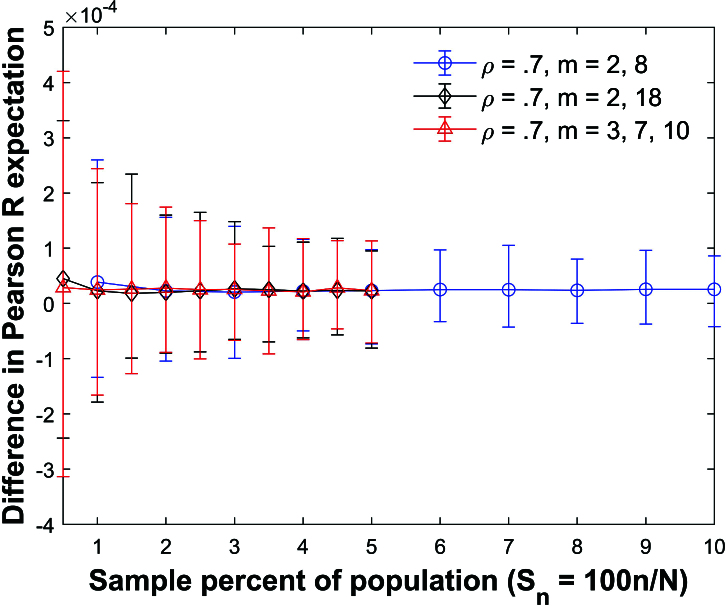
Plot of difference $\left(E\,\left(R_{\mathrm{sim}}\right)-E\,\left(R\right)\right)$ using a population size N=10,000 sampled from a bivariate distribution with ρ=0.7 plotted against the sample percent of the population $S_{n}=100\,\frac{n}{N}$ using groups of mixed size.

**Figure 20:**
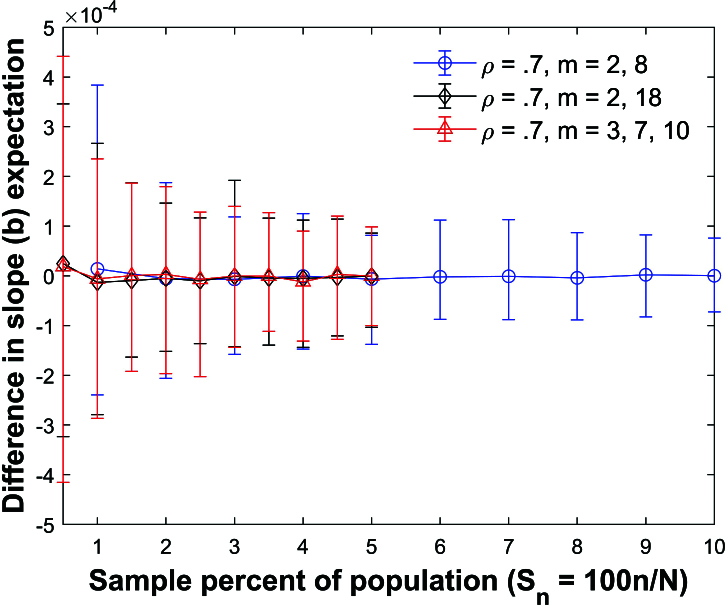
Plot of difference $\left(E\,\left(b_{\mathrm{sim}}\right)-E\,\left(b\right)\right)$ using a population size N=10,000 sampled from a bivariate distribution with ρ=0.7 plotted against the sample percent of the population $S_{n}$ using groups of mixed size.

**Figure 21:**
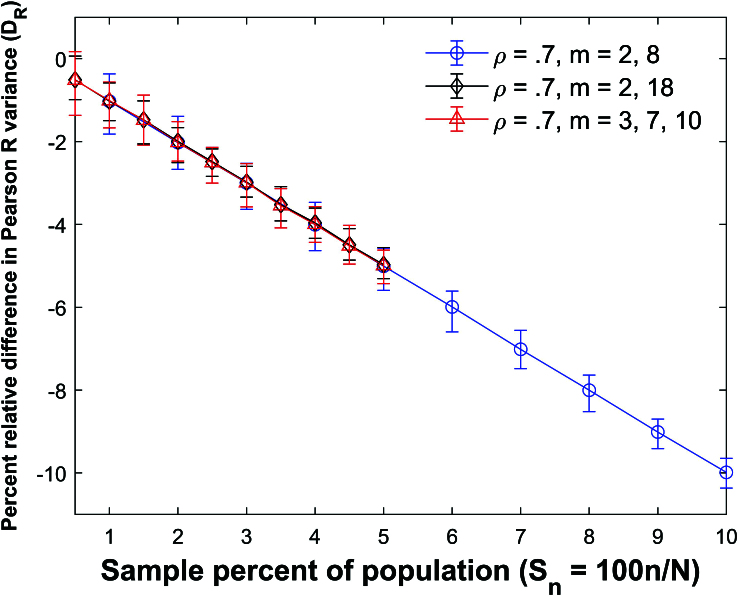
Plot of percent relative difference $D_{R}=100\,\left(\frac{\mathrm{Var}\,\left(R_{\mathrm{sim}}\right)-\mathrm{Var}\,\left(R\right)}{\mathrm{Var}\,\left(R\right)}\right)$ using a population size N=10,000 sampled from a bivariate distribution with ρ=0.7 plotted against the sample percent of the population $S_{n}$ using groups of mixed size. The percent relative difference is closely approximated with the linear equation $D_{R}=-S_{n}$ .

**Figure 22:**
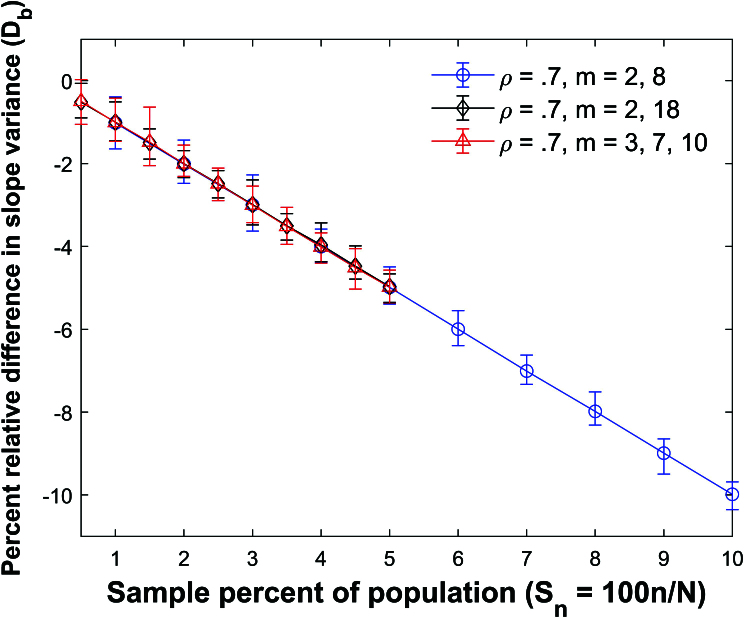
Plot of percent relative difference $D_{b}=100\,\left(\frac{\mathrm{Var}\,\left(b_{\mathrm{sim}}\right)-\mathrm{Var}\,\left(b\right)}{\mathrm{Var}\,\left(b\right)}\right)$ using a population size N=10,000 sampled from a bivariate distribution with ρ=0.7 plotted against the sample percent of the population $S_{n}$ using groups of mixed size. The percent relative difference can be described by the linear equation $D_{b}=-S_{n}$ .

In Figures 19–22, we study the behavior of groups of mixed size. In all the figures, the population size is N=10,000 and the samples are generated from a bivariate distribution with ρ=0.7 , $\left(\mu_{x},\mu_{y}\right)=\left(0,0\right)$ , and $\left(\sigma_{x},\sigma_{y}\right)=\left(1,1\right)$ . Figures 19 and 20 plot the difference $\left(E\,\left(R_{\mathrm{sim}}\right)-E\,\left(R\right)\right)$ and $\left(E\,\left(b_{\mathrm{sim}}\right)-E\,\left(b\right)\right)$ respectively for three types of mixed groups:

(1)
m=2 and m=8 ,
(1)
m=2 and m=18 ,
(1)
m=3 , m=7 , and m=10 .

There are equal amounts of each group size in each sample. The plot symbol shows the average difference and the error bars show the maximum and minimum errors using 112 different populations. The differences in the expectation values remain small. However, the size of the error bars increases as the sample percent of the population $S_{n}$ decreases.

Figure 21 and Figure 22 plot $D_{R}=100\,\left(\frac{\mathrm{Var}\,\left(R_{\mathrm{sim}}\right)-\mathrm{Var}\,\left(R\right)}{\mathrm{Var}\,\left(R\right)}\right)$ and $D_{b}=100\,\left(\frac{\mathrm{Var}\,\left(b_{\mathrm{sim}}\right)-\mathrm{Var}\,\left(b\right)}{\mathrm{Var}\,\left(b\right)}\right)$ respectively for the mixed groups. We observe again that $D_{R}=-S_{n}$ and $D_{b}=-S_{n}$ are good approximations to the plots of $D_{R}$ versus $S_{n}$ and $D_{b}$ versus $S_{n}$ . The absolute errors in approximating the plot of $D_{R}$ vs $S_{n}$ with the line $D_{R}=-S_{n}$ and $D_{b}$ vs $S_{n}$ with the line $D_{b}=-S_{n}$ are all less than 0.05% .

### 4.5 Non-normal populations

In this subsection, we consider random samples drawn from non-normal distributions. In all Figures 23–35, the population size is N=10,000 and ρ=0.7 . Figure 23, Figure 24, and Figure 25 plot the difference $\left(E\,\left(R_{\mathrm{sim}}\right)-E\,\left(R\right)\right)$ sampled from a uniform ( f⁢(x)=f⁢(y)=1 , 0≤x,y≤1 ), an exponential

$$f\,\left(x\right)=e^{-x},f\,\left(y\right)=e^{-y},0\leq x,y<\infty ,$$

and a bimodal distribution

$$f\,\left(x\right)=\frac{e^{-{\left(x+1\right)}^{2}}+e^{-{\left(x-1\right)}^{2}}}{2\,\sqrt{2\,\pi }},f\,\left(y\right)=\frac{e^{-\frac{y^{2}}{2}}}{\sqrt{2\,\pi }}$$

respectively. The plot symbol shows the average difference and the error bars show the maximum and minimum differences using 112 different populations. The average differences are small but their absolute values increase as the sample percent of the population $S_{n}$ decreases.

**Figure 23:**
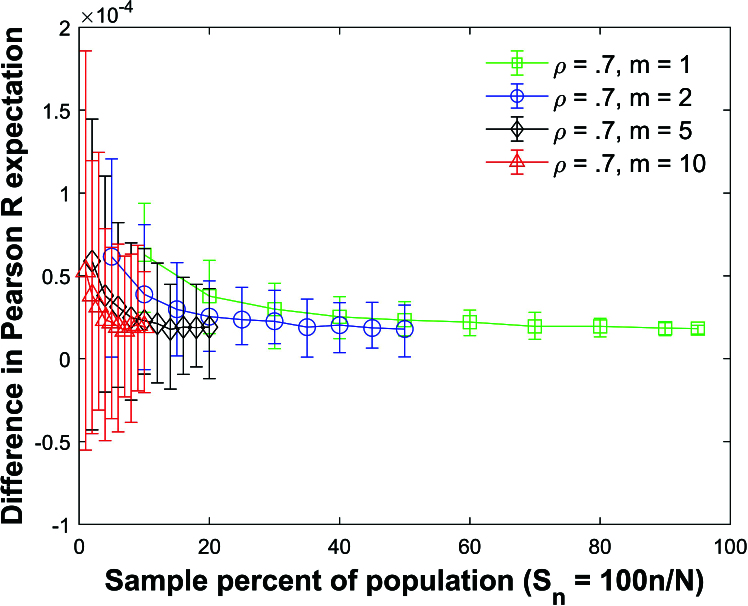
Plot of difference $\left(E\,\left(R_{\mathrm{sim}}\right)-E\,\left(R\right)\right)$ using a population size N=10,000 sampled from a **uniform** distribution with ρ=0.7 plotted against the sample percent of the population $S_{n}=100\,\frac{n}{N}$ . The plot symbol shows the average difference and the error bars show the maximum and minimum differences using 112 different populations of size N=10,000 .

**Figure 24:**
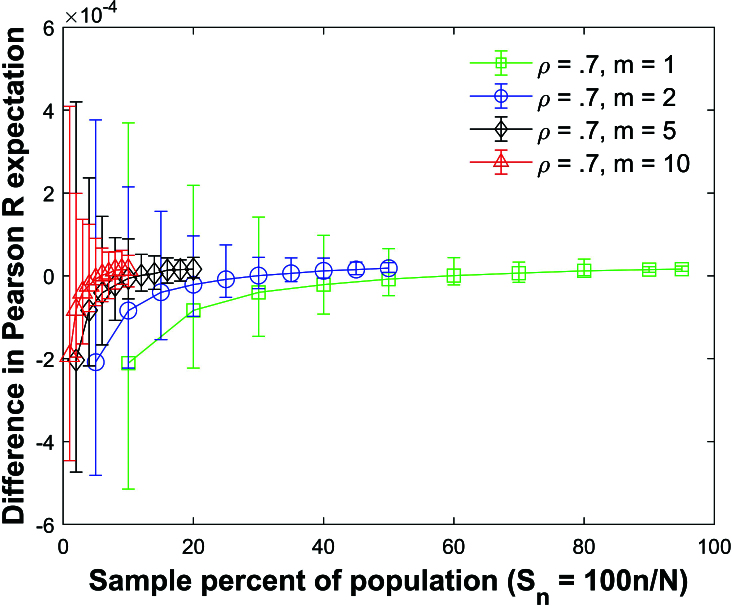
Plot of difference $\left(E\,\left(R_{\mathrm{sim}}\right)-E\,\left(R\right)\right)$ using a population size N=10,000 sampled from an **exponential** distribution with ρ=0.7 plotted against the sample percent of the population $S_{n}$ .

**Figure 25:**
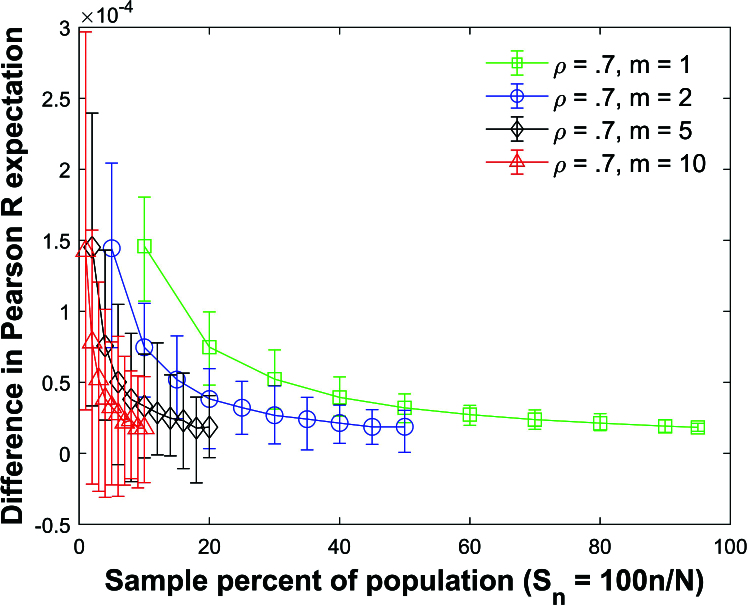
Plot of difference $\left(E\,\left(R_{\mathrm{sim}}\right)-E\,\left(R\right)\right)$ using a population size N=10,000 sampled from a **bimodal** distribution with ρ=0.7 plotted against the sample percent of the population $S_{n}$ .

**Figure 26:**
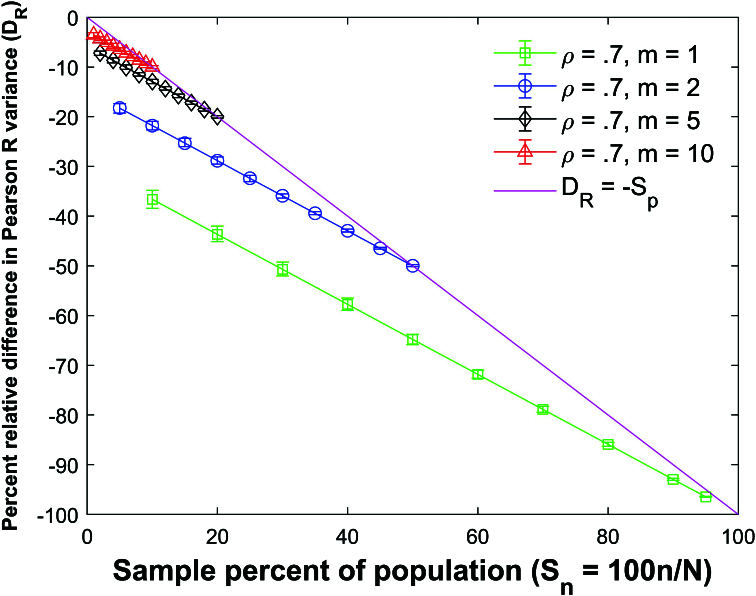
Plot of percent relative difference $D_{R}=100\,\left(\frac{\mathrm{Var}\,\left(R_{\mathrm{sim}}\right)-\mathrm{Var}\,\left(R\right)}{\mathrm{Var}\,\left(R\right)}\right)$ using a population size N=10,000 sampled from a **uniform** distribution with ρ=0.7 plotted against the sample percent of the population $S_{n}$ .

**Figure 27:**
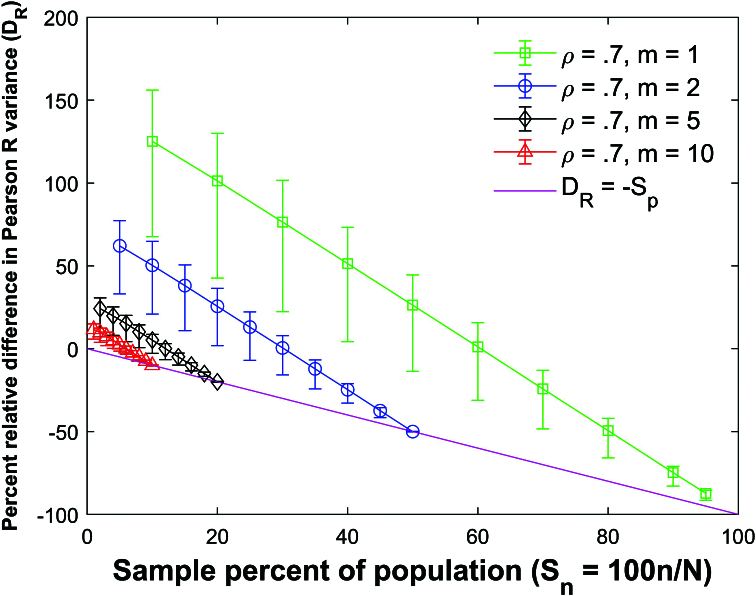
Plot of percent relative difference $D_{R}=100\,\left(\frac{\mathrm{Var}\,\left(R_{\mathrm{sim}}\right)-\mathrm{Var}\,\left(R\right)}{\mathrm{Var}\,\left(R\right)}\right)$ using a population size N=10,000 sampled from an **exponential** distribution with ρ=0.7 plotted against the sample percent of the population $S_{n}$ .

**Figure 28:**
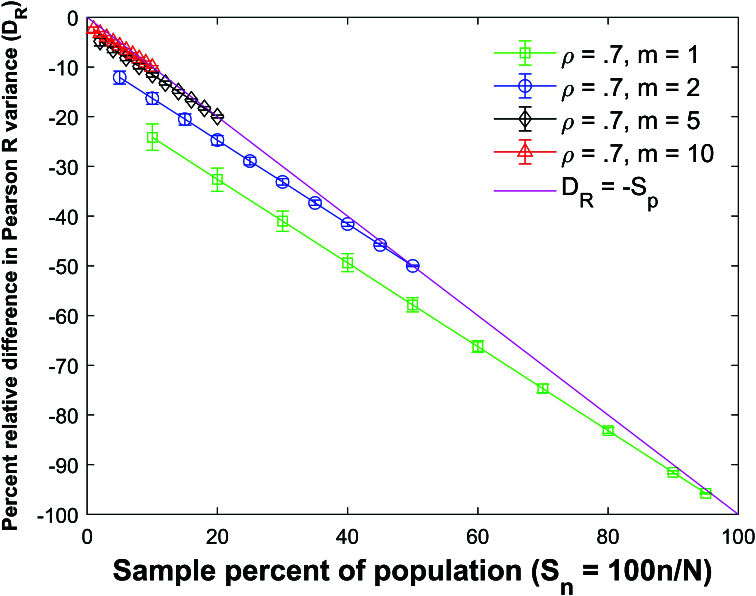
Plot of percent relative difference $D_{R}=100\,\left(\frac{\mathrm{Var}\,\left(R_{\mathrm{sim}}\right)-\mathrm{Var}\,\left(R\right)}{\mathrm{Var}\,\left(R\right)}\right)$ using a population size N=10,000 sampled from a **bimodal** distribution with ρ=0.7 plotted against the sample percent of the population $S_{n}$ .

**Figure 29:**
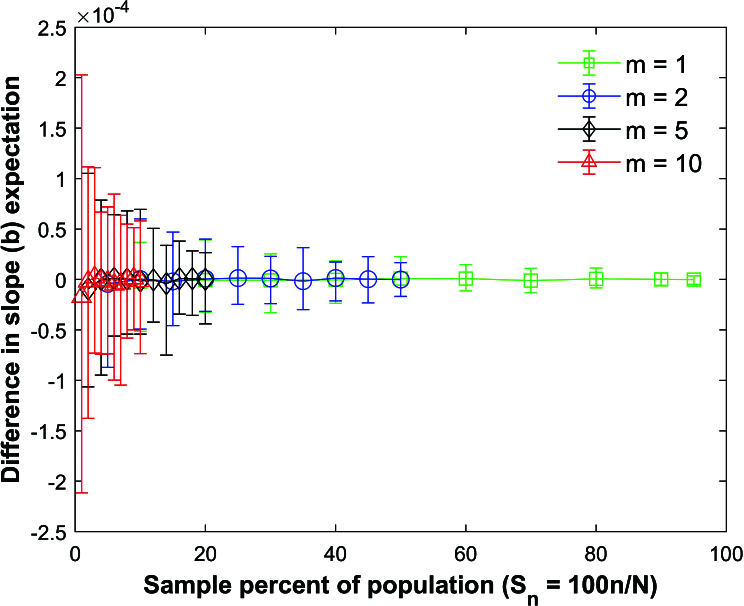
Plot of difference $\left(E\,\left(b_{\mathrm{sim}}\right)-E\,\left(b\right)\right)$ using a population size N=10,000 sampled from a **uniform** distribution with ρ=0.7 plotted against the sample percent of the population $S_{n}$ .

**Figure 30:**
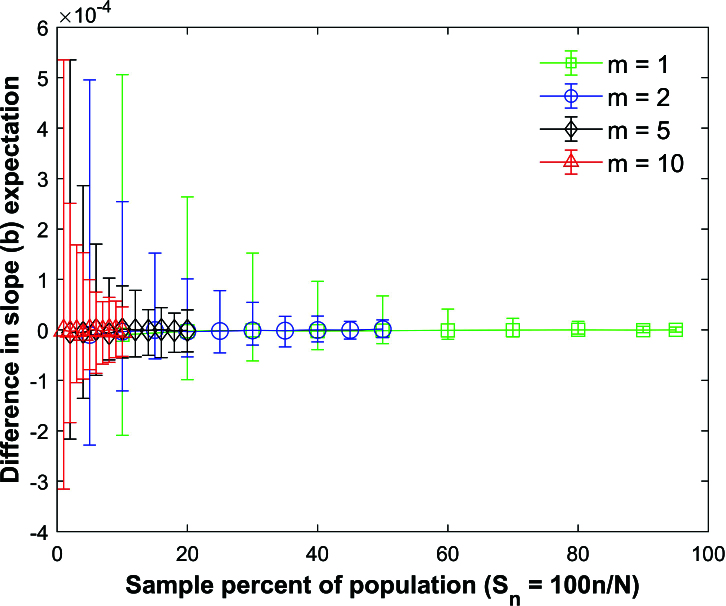
Plot of difference $\left(E\,\left(b_{\mathrm{sim}}\right)-E\,\left(b\right)\right)$ using a population size N=10,000 sampled from an **exponential** distribution with ρ=0.7 plotted against the sample percent of the population $S_{n}$ .

**Figure 31:**
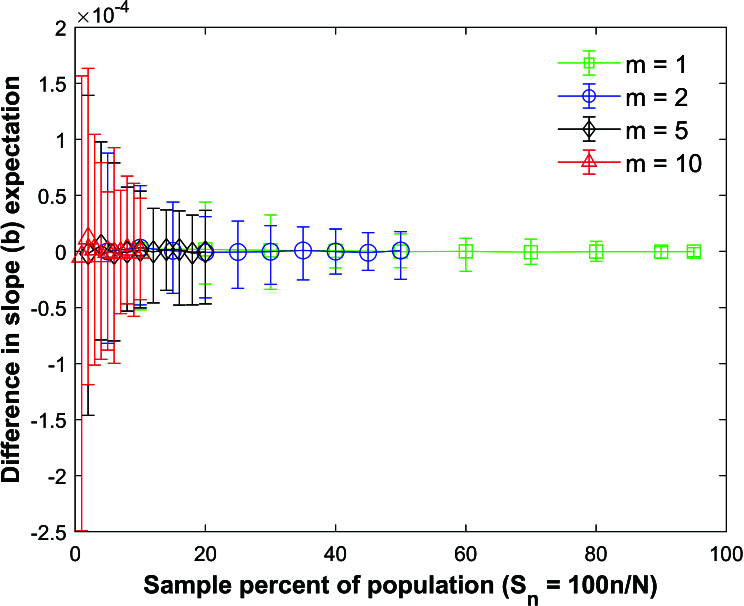
Plot of difference $\left(E\,\left(b_{\mathrm{sim}}\right)-E\,\left(b\right)\right)$ using a population size N=10,000 sampled from a **bimodal** distribution with ρ=0.7 plotted against the sample percent of the population $S_{n}$ .

**Figure 32:**
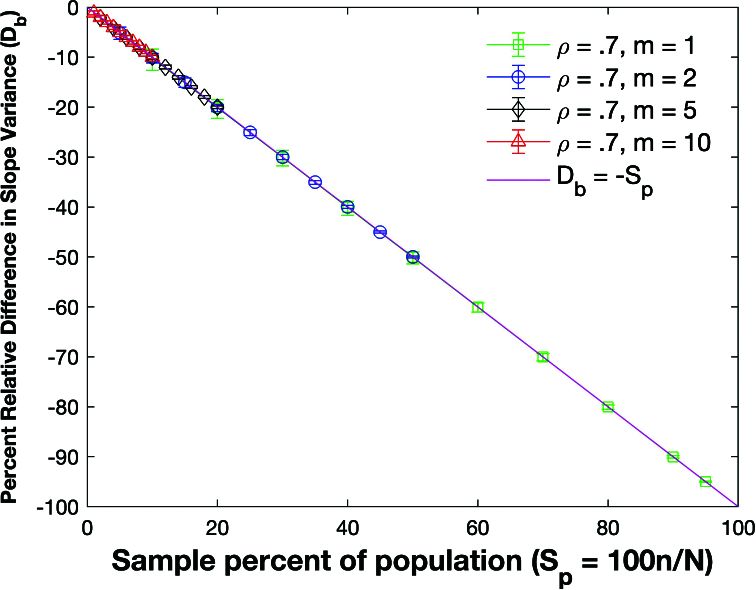
Plot of percent relative difference $D_{b}=100\,\left(\frac{\mathrm{Var}\,\left(b_{\mathrm{sim}}\right)-\mathrm{Var}\,\left(b\right)}{\mathrm{Var}\,\left(b\right)}\right)$ using a population size N=10,000 sampled from a **uniform** distribution with ρ=0.7 plotted against the sample percent of the population $S_{n}$ . The absolute errors in approximating the plot of $D_{b}$ vs $S_{n}$ with the line $D_{b}=-S_{n}$ are all less than 0.18 %.

**Figure 33:**
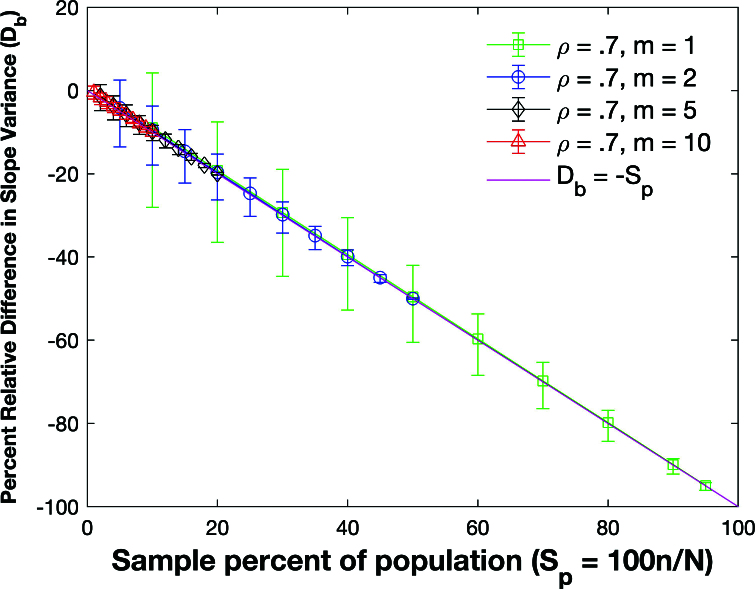
Plot of percent relative difference $D_{b}=100\,\left(\frac{\mathrm{Var}\,\left(b_{\mathrm{sim}}\right)-\mathrm{Var}\,\left(b\right)}{\mathrm{Var}\,\left(b\right)}\right)$ using a population size N=10,000 sampled from an **exponential** distribution with ρ=0.7 plotted against the sample percent of the population $S_{n}$ . The absolute errors in approximating the plot of $D_{b}$ vs $S_{n}$ with the line $D_{b}=-S_{n}$ are all less than 1.2 %.

**Figure 34:**
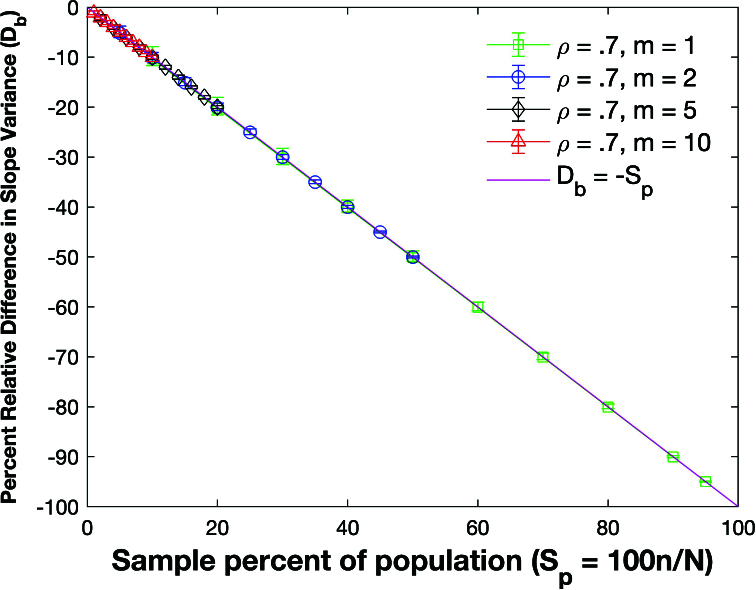
Plot of percent relative difference $D_{b}=100\,\left(\frac{\mathrm{Var}\,\left(b_{\mathrm{sim}}\right)-\mathrm{Var}\,\left(b\right)}{\mathrm{Var}\,\left(b\right)}\right)$ using a population size N=10,000 sampled from a **bimodal** distribution with ρ=0.7 plotted against the sample percent of the population $S_{n}$ . The absolute errors in approximating the plot of $D_{b}$ vs $S_{n}$ with the line $D_{b}=-S_{n}$ are all less than 0.35 %.

**Figure 35:**
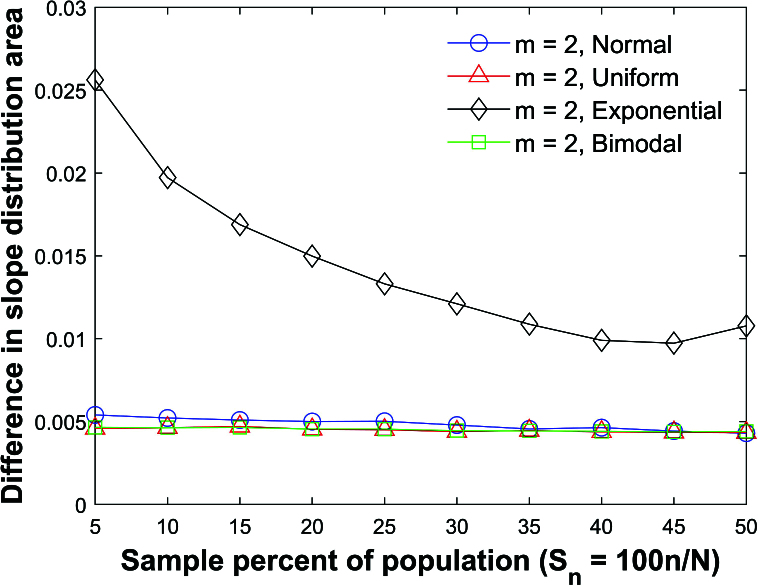
Plot of difference $\int \left|h_{\mathrm{sim}}\,\left(b\right)-h\,\left(b\right)\right|\,𝑑b$ , where $h_{\mathrm{sim}}\,\left(b\right)$ is the distribution formed by the Monte Carlo simulation, h⁢(b) is the slope distribution (2.9), and $\left\{\left(x_{i},y_{i}\right)\right\}$ are sampled from normal, uniform, exponential, and bimodal distributions.

Figure 26, Figure 27, and Figure 28 plot $D_{R}=100\,\left(\frac{\mathrm{Var}\,\left(R_{\mathrm{sim}}\right)-\mathrm{Var}\,\left(R\right)}{\mathrm{Var}\,\left(R\right)}\right)$ sampled from a uniform, exponential, and bimodal distribution respectively. The magenta line $D_{R}=-S_{n}$ is included as a reference (as it served as a good approximation when sampling from normal distributions). The plots of $D_{R}$ vs $S_{n}$ appear linear. However, unlike samples from normal distributions, $D_{R}$ is a function of **both** the sample percent of the population $S_{n}$ and the group size *m*. The line for m=10 does seem to match the $D_{R}=-S_{n}$ line better compared to smaller values of *m* due to the central limit theorem. Interestingly, we note that for m>1 the plots intersect the line $D_{R}=-S_{n}$ when $m\,S_{n}=100$ . We also tried generating Var⁢(R) by sampling **without** replacement for each non-normal population and found that $D_{R}$ still could not be approximated with $D_{R}=-S_{n}$ .

Figure 29, Figure 30, and Figure 31 plot the difference $\left(E\,\left(b_{\mathrm{sim}}\right)-E\,\left(b\right)\right)$ sampled from a uniform, exponential, and bimodal distribution respectively. Since the difference is small, E⁢(b) computed from (2.10) is a good approximation for the expectation of the sampling distribution.

Figure 32, Figure 33, and Figure 34 plot $D_{b}=100\,\left(\frac{\mathrm{Var}\,\left(b_{\mathrm{sim}}\right)-\mathrm{Var}\,\left(b\right)}{\mathrm{Var}\,\left(b\right)}\right)$ sampled from a uniform, exponential, and bimodal distribution respectively; Var⁢(b) is computed using (2.11). The magenta line $D_{b}=-S_{n}$ is included as a reference. Remarkably, $D_{b}$ is only a function of $S_{n}$ and does not appear to depend on the group size *m* (unlike $D_{R}$ ). We note that Goodman’s [7] method relies on the premise that regression coefficients are less easily affected by aggregation than correlation [10].

Figure 35 plots the area difference $\int \left|h_{\mathrm{sim}}\,\left(b\right)-h\,\left(b\right)\right|\,𝑑b$ , where $h_{\mathrm{sim}}\,\left(b\right)$ is the distribution formed by the Monte Carlo simulation, h⁢(b) is the slope distribution (2.9), and $\left\{\left(x_{i},y_{i}\right)\right\}$ are sampled without replacement from normal, uniform, exponential, and bimodal distributions. The variance of h⁢(b) is modified by adjusting the value of *n* in order to match the variance of the line $D_{b}=-S_{n}$ . The largest area difference is generated with the exponential distribution, but all differences are less than 0.026. Figure 35 shows that the simulated distributions can be approximated with h⁢(b) for these populations types.

### 4.6 Multiple regression results

**Figure 36:**
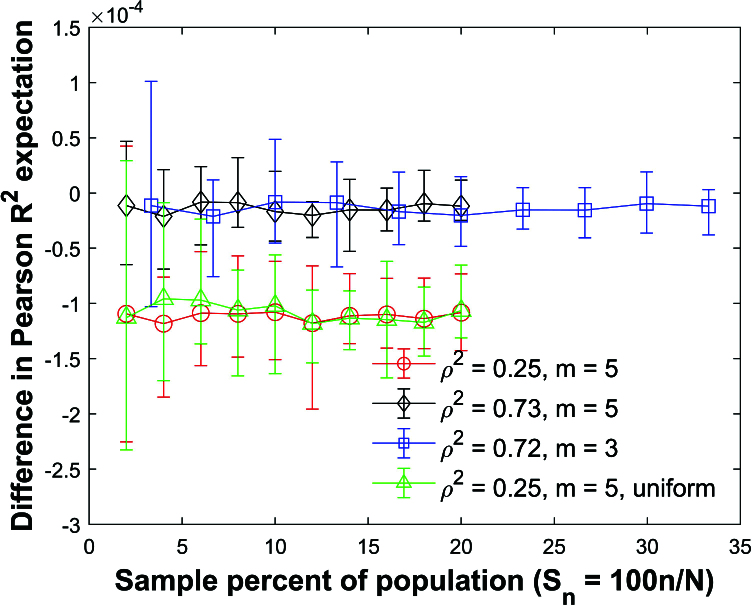
Plot of difference $\left(E\,\left(R_{\mathrm{sim}}^{2}\right)-E\,\left(R^{2}\right)\right)$ using a population size N=10,000 sampled from a bivariate distribution with $\rho^{2}=.25$ and m=5 , $\rho^{2}=0.73$ and m=5 , and $\rho^{2}=0.72$ and m=3 . A plot of $\rho^{2}=0.25$ and m=5 is also included which was sampled from a uniform distribution. The symbol shows the average difference and the error bars show the maximum and minimum differences using 112 different populations. The expectation $E\,\left(R^{2}\right)$ is computed using (2.13) and $E\,\left(R_{\mathrm{sim}}^{2}\right)$ is generated using a Monte Carlo simulation in which a million samples are selected without replacement using different sample sizes *n* (displayed along the horizontal axis using the sample percent of the population $S_{n}=100\,\frac{n}{N}$ ).

We move now to multiple regression simulations. Figure 36 plots the difference $\left(E\,\left(R_{\mathrm{sim}}^{2}\right)-E\,\left(R^{2}\right)\right)$ using a population size N=10,000 sampled from a normal distribution and correlated using Cholesky decomposition with $\rho^{2}=0.25$ $\rho^{2}=0.73$ , and $\rho^{2}=0.72$ . For $\rho^{2}=0.25$ and m=5 , the correlation matrix 𝐂 has the following off-diagonal elements: $R_{z\,x}=C_{1,2}=C_{2,1}=-0.5$ , $R_{z\,y}=C_{1,3}=C_{3,1}=0.1$ , $R_{x\,y}=C_{2,3}=C_{3,2}=-0.055$ . We sample from both a normal and uniform distribution when $\rho^{2}=0.25$ . For ρ=0.73 and m=5 , the correlation matrix has the following off-diagonal elements: $R_{z\,x}=C_{1,2}=C_{2,1}=0.7$ , $R_{z\,y}=C_{1,3}=C_{3,1}=0.8$ , $R_{x\,y}=C_{2,3}=C_{3,2}=0.56$ . For ρ=0.72 and m=3 , the correlation matrix has the following off-diagonal elements: $R_{z\,x}=C_{1,2}=C_{2,1}=-0.3$ , $R_{z\,y}=C_{1,3}=C_{3,1}=0.7$ , $R_{x\,y}=C_{2,3}=C_{3,2}=-0.2$ . The symbol shows the average difference and the error bars show the maximum and minimum differences using 112 different populations. The expectation $E\,\left(R^{2}\right)$ is computed using (2.13) and $E\,\left(R_{\mathrm{sim}}^{2}\right)$ is generated using a Monte Carlo simulation in which a million samples are randomly selected without replacement. The differences $\left(E\,\left(R_{\mathrm{sim}}^{2}\right)-E\,\left(R^{2}\right)\right)$ are small but the size of the error bars increase as $S_{n}$ decreases.

**Figure 37:**
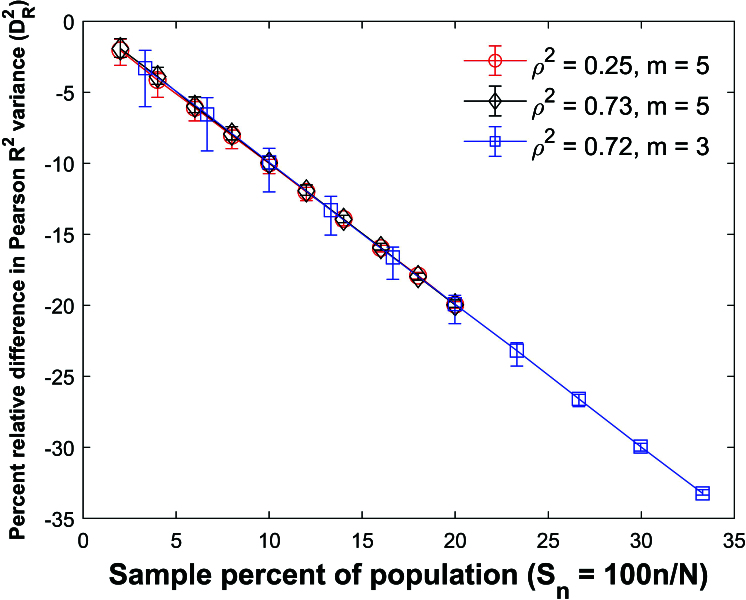
Plot of percent relative difference $D_{R}^{2}=100\,\left(\frac{\mathrm{Var}\,\left(R_{\mathrm{sim}}^{2}\right)-\mathrm{Var}\,\left(R^{2}\right)}{\mathrm{Var}\,\left(R^{2}\right)}\right)$ using a population size N=10,000 sampled from a bivariate distribution with $\rho^{2}=0.25$ and m=5 , $\rho^{2}=0.73$ and m=5 , and $\rho^{2}=0.72$ and m=3 . The symbol shows the average difference and the error bars show the maximum and minimum differences using 112 different populations. The variance $\mathrm{Var}\,\left(R^{2}\right)$ is computed using (2.14) and $\mathrm{Var}\,\left(R_{\mathrm{sim}}^{2}\right)$ is generated using a Monte Carlo simulation in which a million samples are selected without replacement.

**Figure 38:**
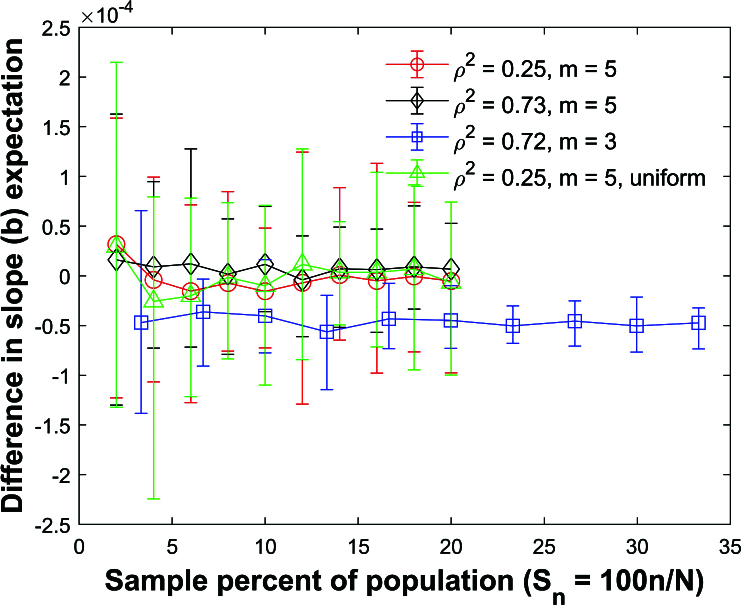
Plot of difference $\left(E\,\left(b_{\mathrm{sim}}\right)-E\,\left(b\right)\right)$ using a population size N=10,000 sampled from a bivariate distribution with $\rho^{2}=0.25$ and m=5 , $\rho^{2}=0.73$ and m=5 , and $\rho^{2}=0.72$ and m=3 . A plot of $\rho^{2}=0.25$ and m=5 is also included which was sampled from a uniform distribution. The symbol shows the average difference and the error bars show the maximum and minimum differences using 112 different populations. The expectation E⁢(b) is generated using a Monte Carlo simulation in which a million samples are selected **with** replacement and $E\,\left(b_{\mathrm{sim}}\right)$ is generated using a Monte Carlo simulation in which a million samples are selected without replacement.

**Figure 39:**
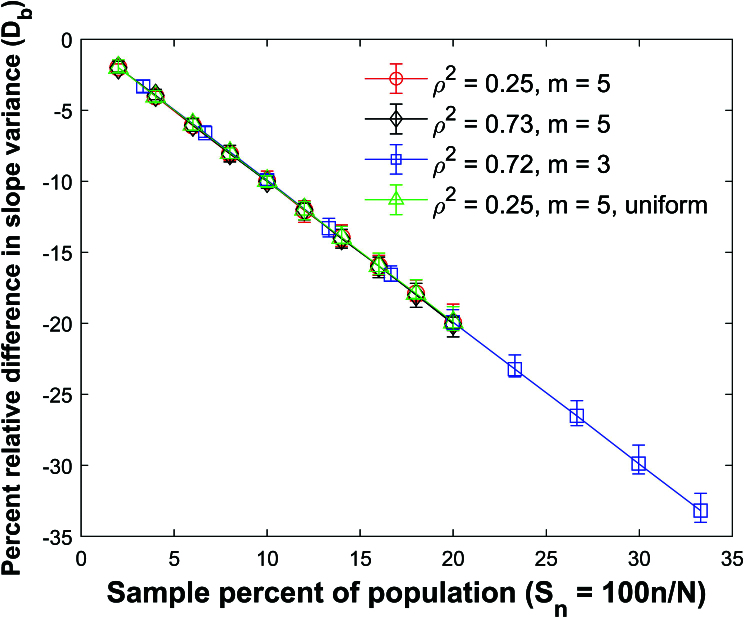
Plot of percent relative difference $D_{b}=100\,\left(\frac{\mathrm{Var}\,\left(b_{\mathrm{sim}}\right)-\mathrm{Var}\,\left(b\right)}{\mathrm{Var}\,\left(b\right)}\right)$ using a population size N=10,000 sampled from a bivariate distribution with $\rho^{2}=0.25$ and m=5 , $\rho^{2}=0.73$ and m=5 , and $\rho^{2}=0.72$ and m=3 . A plot of $\rho^{2}=0.25$ and m=5 is also included which was sampled from a uniform distribution. The symbol shows the average difference and the error bars show the maximum and minimum differences using 112 different populations. The variance Var⁢(b) is generated using a Monte Carlo simulation in which a million samples were selected **with** replacement and $\mathrm{Var}\,\left(b_{\mathrm{sim}}\right)$ is generated using a Monte Carlo simulation in which million samples are selected without replacement.

Figure 37 plots the percent relative difference $D_{R}^{2}=100\,\left(\frac{\mathrm{Var}\,\left(R_{\mathrm{sim}}^{2}\right)-\mathrm{Var}\,\left(R^{2}\right)}{\mathrm{Var}\,\left(R^{2}\right)}\right)$ using a population size N=10,000 sampled from a bivariate distribution under the same conditions as Figure 36. The symbol shows the average difference and the error bars show the maximum and minimum differences using 112 different populations. The variance $\mathrm{Var}\,\left(R^{2}\right)$ is generated using (2.14) and $\mathrm{Var}\,\left(R_{\mathrm{sim}}^{2}\right)$ is generated using a Monte Carlo simulation in which a million samples are randomly selected without replacement. The percent relative difference can be approximated with the line $D_{R}^{2}=-S_{n}$ .

Figure 38 plots the difference $\left(E\,\left(b_{\mathrm{sim}}\right)-E\,\left(b\right)\right)$ using a population size N=10,000 sampled from a bivariate distribution with $\rho^{2}=0.25$ , $\rho^{2}=0.73$ , and ρ=0.72 using the slope *b* between *z* and *x*. We also include a plot of $\rho^{2}=0.25$ which was created by sampling from a uniform distribution. The expectation E⁢(b) is generated using a Monte Carlo simulation in which a million samples are selected **with** replacement and $E\,\left(b_{\mathrm{sim}}\right)$ is generated using a Monte Carlo simulation in which a million samples are selected without replacement. The differences $\left(E\,\left(b_{\mathrm{sim}}\right)-E\,\left(b\right)\right)$ are small.

Figure 39 plots the percent relative difference $D_{b}=100\,\left(\frac{\mathrm{Var}\,\left(b_{\mathrm{sim}}\right)-\mathrm{Var}\,\left(b\right)}{\mathrm{Var}\,\left(b\right)}\right)$ under the same conditions as Figure 38 using the slope *b* between *z* and *x*. We also include a plot of $\rho^{2}=0.25$ which was created by sampling from a uniform distribution. The variance Var⁢(b) is generated using a Monte Carlo simulation in which a million samples are selected **with** replacement and $\mathrm{Var}\,\left(b_{\mathrm{sim}}\right)$ is generated using a Monte Carlo simulation in which a million samples are selected without replacement. The percent relative difference can be approximated with the line $D_{b}=-S_{n}$ even for the case where samples were drawn from a uniform distribution.

## 5 Discussion and conclusion

In this article, we perform Monte Carlo simulations to select samples without replacement from finite populations to generate distributions of Pearson *R*, slope *b*, and the coefficient of determination $R^{2}$ in simple and multiple regression contexts. Our Monte Carlo simulations suggest that the expectations of the *R*, *b*, and $R^{2}$ distributions are similar to the expectations of the analytical sampling distributions for normally distributed data for both individual and group averaged data as long as the sample sizes *n* are the same. This observation is also true for groups of mixed sizes and for the three non-normal distributions we tested using simple regression simulations.

However the variances of the *R*, *b*, and $R^{2}$ distributions created without replacement are reduced compared to the variances of the analytical distributions. Our simulations show that the percent relative differences $D_{R}$ , $D_{b}$ , and $D_{R}^{2}$ in the variances can be approximated with the linear equations $D_{R}=-S_{n}$ , $D_{b}=-S_{n}$ , and $D_{R}^{2}=-S_{n}$ , where $S_{n}$ is the sample percent of the population $S_{n}=100\,\frac{n}{N}$ . The group size that is used when selecting the samples does not affect the variances for normally distributed variables. We observe the same results when considering groups of mixed size.

The distribution of the Fisher transformed value of *R* denoted by $R_{z}$ can also be approximated by a normal distribution as the sample size *n* increases when sampling without replacement for both individual and group averaged data. Similar to the other percent relative differences, $D_{z}$ can be approximated with the line $D_{z}=-S_{n}$ .

We also observed that for non-normal distributions, the percent relative differences in the correlation variances $D_{R}$ depend on the sample size and the group size and cannot be approximated with a simple line. Interestingly, in contrast, the percent relative differences in **slope** variances $D_{b}$ **can** be approximated by the line $D_{b}=-S_{n}$ for the non-normal distributions we considered.

Our observations afford another interpretation of the ecological fallacy and suggest that for random samples drawn without replacement from normally distributed finite populations, the correlation coefficients and linear regression slopes will be selected from approximately the same sampling distribution regardless of the group size *m* as long as the sample size *n* is the same.

## A Appendix: Fortran code

The parallel Fortran code we use to run the Monte Carlo simulations evenly divides the 112 populations among the processors. Each processor then develops the Pearson R and slope distributions using its share of the populations for different values of group size *m* and different sample percents of the population $S_{n}$ .

Each processor finds the maximum, minimum, and average difference of the expectation and variance of each distribution from the analytical value for its share of the populations. Then the maximum, minimum, and average difference of the expectation and variance are shared among all the processors, to find the maximum, minimum, and average difference for all populations which is stored on the first processor.

We have developed two versions of the code: an MPI version and a Coarray Fortran version. MPI uses the MPI_REDUCE calls for communication and the Coarray Fortran uses the co_sum, co_max, and co_min calls for communication.
